# Global trends and hotspots in macrophage research related to hypertension from 2015-2024: bibliometric research and visualization analysis

**DOI:** 10.3389/fimmu.2025.1501432

**Published:** 2025-03-05

**Authors:** Xuefei Wang, Yemao Chai, Ye Dou, Xinyi Li, Fanghe Li, Kuo Gao

**Affiliations:** Beijing University of Chinese Medicine, Beijing, China

**Keywords:** macrophage, hypertension, bibliometric, visualization, hotspots

## Abstract

**Background:**

Hypertension continues to be a global health and economic burden, conventionally characterized by a chronic inflammatory state. Macrophages are critical for the initiation, progression and manifestation of hypertension. As studies on the relationship between macrophages and hypertension increase substantially, identifying critical research areas and unraveling potential interaction mechanisms become increasingly essential.

**Methods:**

Articles associated with hypertension and macrophages in recent 10 years were retrieved from the Web of Science Core Collection for analysis, using Microsoft Excel, VOSviewer, CiteSpace and Scimago Graphica.

**Results:**

After excluding studies that did not meet inclusive standard based on time (2015-2024) and type (article or reviews), 2,013 original articles related to macrophages associated with hypertension were included. The number of publications has been increasing annually. These records consisted of 2,013 English language papers published in 351 journals by 315 institutions or regions from 83 countries/regions between 2015 and 2024. We analyzed the co-cited references clusters to objectively outline the current state of research, including the regulatory mechanisms of hypertension, diseases related to hypertension, and the lifestyle factor. Inflammation remains one of the most popular research hot-spot. The most popular publishing journal in this field is PLOS ONE and the most prolific writer is Li, Hui-Hua. The primary keywords cluster in this field is inflammation, with the highest occurrences and TLS among the top 10 keywords.

**Conclusion:**

These comprehensive and visualized bibliometric results summarized the significant findings in macrophage-related hypertension studies over the past 10 years. Macrophages appear to be effective in the treatment of hypertension as potential targets, but further research is needed to clarify the specific pathophysiological mechanisms involved.

## 1 Introduction

Hypertension is a major factor in the global health and economic burden, affecting over 1 billion adults worldwide (1). Despite the development of various anti-hypertensive drugs, the number of patients continues to rise. The immune system is now recognized as a key component of the multifactorial etiology of hypertension and its related organ damage (2). Hypertension has been conventionally described as a chronic inflammatory state, with inflammatory dysregulation and immune activation closely linked to the progression of hypertension (3). Both innate and adaptive immune cells are involved in a complex interaction that regulates inflammatory and immune mechanisms of hypertension. Innate immunity serves as a swift, nonspecific response to various external stimuli, typically regarded as the early phase of inflammation (4). “Classical” monocytes are prevalent and exhibit a strong reactivity to inflammatory cues, enabling them to penetrate tissues and differentiate into macrophages (5). The essential involvement of immune cells, cytokines, and chemokines in the initiation and development of hypertension is well established. Various immune cells, including T cells, monocytes, macrophages, dendritic cells, B cells, and natural killer (NK) cells, are associated with hypertension (6). Factors such as neoantigens, the NLRP3 inflammasome, heightened sympathetic outflow, and specific cytokines (including IL-6, IL-7, IL-15, IL-18, and IL-21), along with a high-salt diet, can promote immune activation in hypertension (7). When activated, these immune cells migrate to specific tissues and organs, including the arteries (notably in the perivascular fat and adventitia), kidneys, and the brain, leading to critical damage and elevated blood pressure (8). As fundamental innate immune cells, macrophages, along with dendritic cells and natural killer (NK) cells, are among the first responders activated in the onset of hypertension, prompting the release of pro-inflammatory cytokines and chemokines (9). Macrophages play a pivotal role in the inflammatory response and act as the primary source of inflammatory cytokines (10).

As the most prevalent and widespread immune cell, macrophages have a fundamental impact on a variety of organ systems implicated in hypertension (11). Their distinctive position enables them to serve as essential mediators among immune components, and their remarkable adaptability allows them to thrive in different environments (12). This adaptability is showcased through a complex system of functional and phenotypic differentiation, mainly identified by their polarization into pro-inflammatory M1 and anti-inflammatory M2 types (13). Although this classification simplifies the intricate dynamics of macrophage polarization, grasping the unique traits of these subsets is crucial to understanding the complex involvement of macrophages in hypertension pathophysiology (12). M1 macrophages exacerbate vascular remodeling, endothelial dysfunction, and renal impairment by enhancing inflammatory responses and oxidative stress. Conversely, M2 macrophages contribute to hypertension and vascular fibrosis by increasing the accumulation of collagen and elastin within the aorta (14). It was observed that immune monocytes in hypertensive patients have a strong proinflammatory phenotype (15), but the proinflammatory cytokines tumor necrosis factor-α (TNF-α) and IL-1β secreted by M1 macrophages can lead to hypotension by triggering diuresis (16). Research on the polarization status of macrophages in the peritoneal cavity and myocardial tissues of rats has revealed that both abdominal and myocardial tissues in primary hypertension exhibit a higher prevalence of pro-inflammatory macrophages compared to anti-inflammatory (17). In hypertensive states, the secretion of humoral factors triggered by sustained high pressure, irregular blood flow, and the activation of the neuroendocrine system leads to the modification of macrophage phenotypes in the abdominal and myocardial tissues of spontaneously hypertensive rats, subsequently facilitating the progression of hypertension and myocardial remodeling (17, 18). Macrophages are particularly crucial in the initiation, development, and manifestation of hypertension (19). The asynchronous switching of macrophage types at various sites may be due to tissue-resident macrophages adapting to alterations in the local tissue environment by mobilizing macrophages from other origins to inflamed areas (20).

According to the Organization for Economic Co-operation and Development, bibliometrics is defined as the “statistical analysis of written publications, such as books or articles” (21). Different from previous reviews, bibliometrics utilizes mathematical operations and statistical applications to describe or reveal relationships between published works. This method can quantitatively measure the contour distribution, relationships, and clustering within a specific field and has become one of the prevalent techniques to evaluate the reliability, quality, and effect of academic work (22). Up to now, Bibliometrics has been applied in medical fields worldwide to produce seminal and innovative articles.

Over the years, human and animal studies on the relationship between macrophages and hypertension have increased substantially. However, bibliometric analysis in this area remains largely unclear. Further research is needed to examine publications, countries, institutions, journals, authors, and keywords in this field. Therefore, the purpose of this research is to systematically analyze the studies on hypertension related to macrophages, aiming to objectively capture the current trends and hotspots in related area.

## 2 Materials and methods

### 2.1 Search strategies and screening

**Figure 1:**
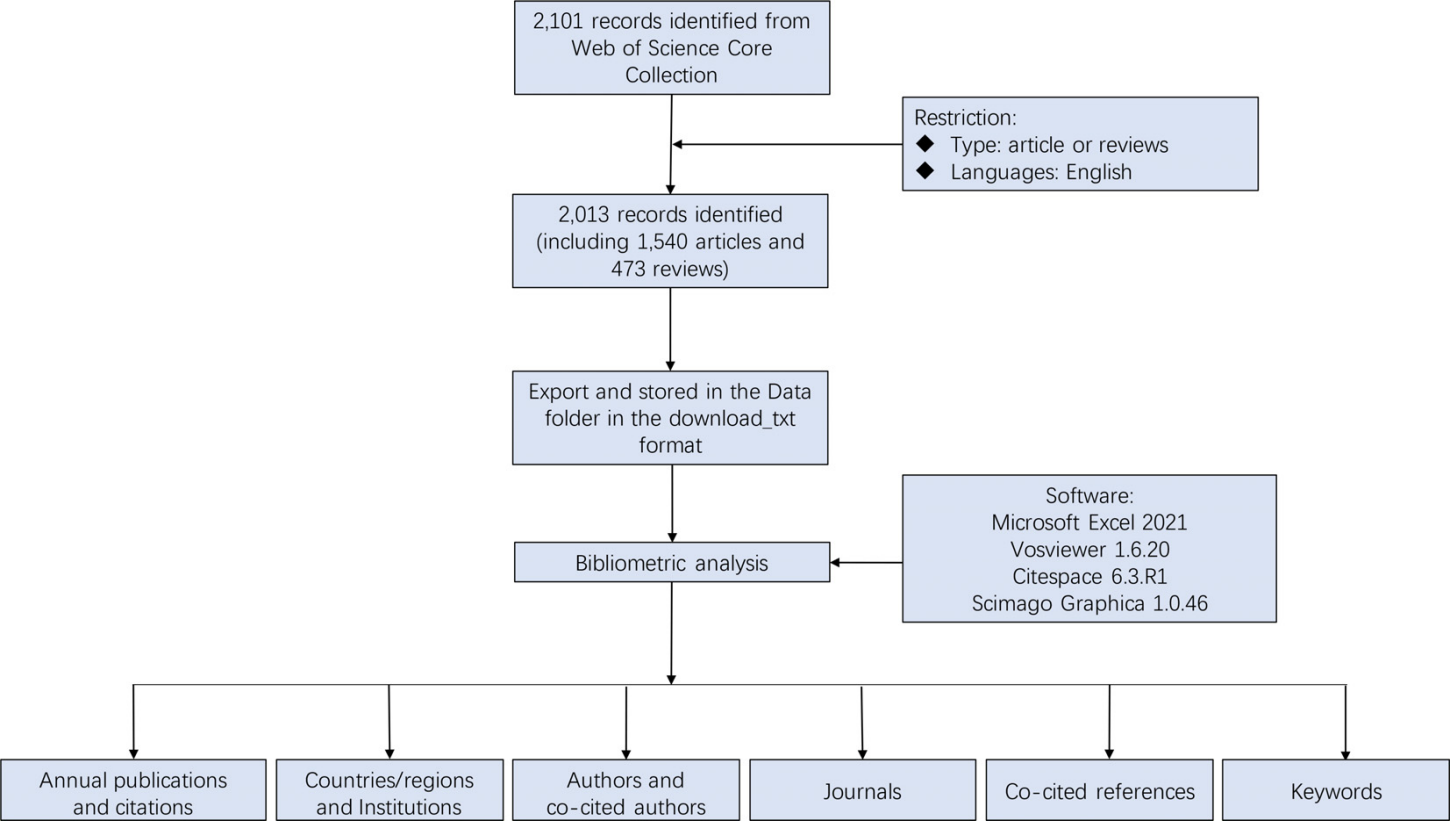
Flowchart of data screening process.

Articles were retrieved and downloaded from the Web of Science Core Collection (WoSCC) which was widely recognized for performing bibliometric analysis. The data included records from Science Citation Index Expanded (SCIE), Social Science Citation Index (SSCI), and Emerging Sources Citation Index (ESCI) databases, and the literature search covered the period from January 1, 2015 to December 31, 2024. The data was accessed and downloaded on January 8, 2025. The search strategy was as follows: (TS=(“Macrophages” OR “Macrophage*” OR “Macrophagy” OR “Macrophagic” OR “Macrophagocyte*”) AND TS=(“Hypertension*” OR “High Blood Pressure*” OR “Hypertensive*” OR “Hyperpiesia*” OR “Hyperpiesis”)) NOT TI=(“Pulmonary Hypertension*” OR “Pulmonary Arterial Hypertension*” OR “Ocular Hypertension*” OR “Hypertensive Retinopath*” OR “Intracranial Hypertension*” OR “Portal Hypertension*”). Among the various publication types, only original articles and reviews were included. **Figure 1** showed the detailed screening process.

### 2.2 Data analysis

The publications were then analyzed using Microsoft Excel 2021, VOSviewer 1.6.20, CiteSpace 6.3.R1 and Scimago Graphica 1.0.46. The general information of references, including the distribution of publication years, countries, organizations, journals, authors, and research fields, was preliminarily analyzed based on the results retrieved from WOSCC.

VOSviewer was utilized to create scientific landscapes and networks focusing on citation, countries, journals, and authors. This software allows for conversion of data related to cooperative relationships into GML format, which can then be imported into Scimago Graphica Beta software to display geographic distribution and country clusters (7). CiteSpace was employed for more comprehensive bibliometric analysis. This tool provided functionalities such as drawing the dual-map overlay of journals, analyzing co-cited authors and references with Citation Bursts, and timeline analyses of keywords and references for a deeper exploration of hot spots and research frontiers. Microsoft Excel was used for sorting and statistical procedures. All the image settings parameters and brief steps are included in the **Supplementary Material**.

## 3 Results

### 3.1 Annual publications and citations growth

**Figure 2:**
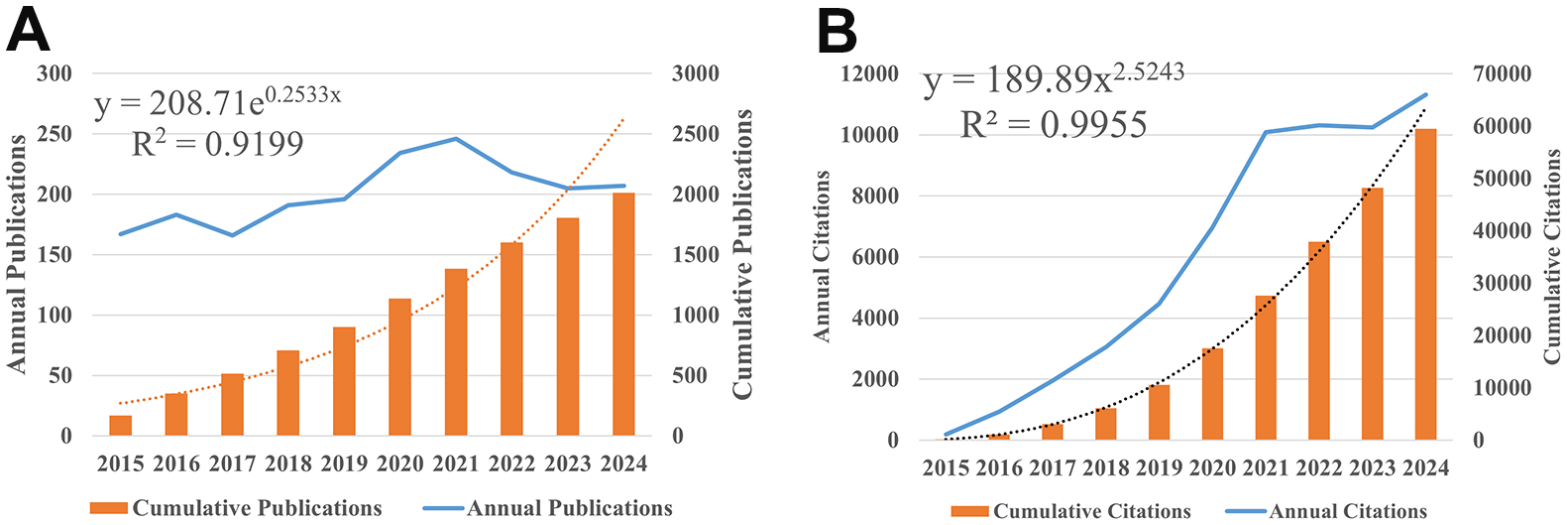
The number of publications in hypertension annually related to Macrophages from 2015 to 2024. **(A)** Annual and cumulative publications quantity. **(B)** Annual and cumulative citation frequency.

After executing the preset retrieval strategy, 2013 published articles and reviews met the inclusion criteria, accumulating 57,721 non-self-cited citations and 104 H index for all retrieved articles, with an average of 29.68 citations per document. The annual number of publications and citation frequency of macrophages research in the field of hypertension were respectively shown in **Figures 2A, B**. Annual publications serve as indicators of the development trends in a certain field, revealing a steady growth trend from 2015 to 2024. A significant surge occurred from 2015 to 2021 on annual citations, especially between 2019 and 2021. Afterwards, a stable high level was maintained from 2021 to 2023, and a new round of significant growth occurred in 2024. This data suggests an increasing interest in studying the correlation between macrophages and hypertension during this period.

### 3.2 Contribution of countries (regions)

**Figure 3:**
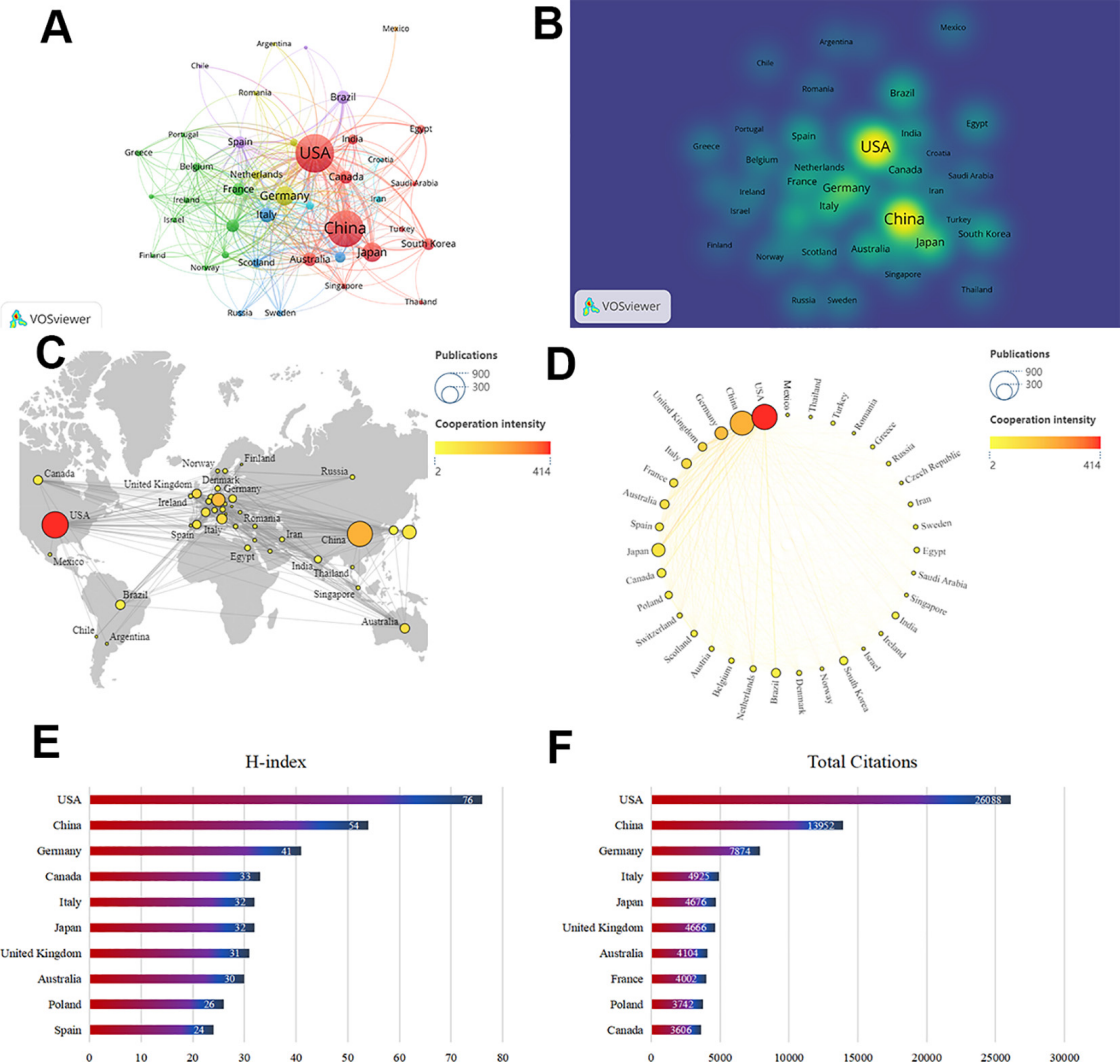
Distribution of countries involved in research on the association between hypertension and Macrophages worldwide. **(A)** The collaborative network map for countries and regions, generated using VOSviewer. **(B)** Visualization of the participation density of all countries and regions. **(C)** Geographical distribution map of global publications. **(D)** Top 30 countries ranked by publication count. **(E)** Top 10 countries with the highest H-index. **(F)** Top 10 most frequently co-cited countries.

The analysis of hypertension research related to macrophages worldwide reveals that 83 countries or regions were involved in the past 10 years. According to **Table 1**, the United States contributed the most publications (641), followed closely by China (579). Together, the US and China accounted for more than half of the total publications (60.25%). As shown the world map in **Figure 3C**, the top 10 countries consist of five European countries, two Asian countries, two North American countries, and one Oceania country. **Figure 3A** elucidated the collaborative connections between countries, while **Figure 3B** showed the publication density across different countries. Consistent with the fact that the USA demonstrated the highest level of international collaboration (as shown in **Figure 3A** and verified by the values in **Figure 3F**), it was also the leading country that published the most articles (**Figure 3B**, the values can be verified in **Table 1**). In other words, the United States positioned itself at the center of this research area. Additionally, the US had its most active international exchange with China (**Figure 3D**). To evaluate the academic contributions of researchers in different countries, we also conducted a statistical analysis of the H-index, as shown in **Figure 3E**. The United States ranks first with an H-index of 76, followed by China (54) and Germany (41). Although the number of publications from China has sharply increased year by year, and the country faces a relatively friendly situation of international cooperation, China still lags behind in average citations among the top 10 countries. This suggests a need for more original research from China.

**Table 1:** Top 10 countries with the most published research on macrophages in hypertension.

Rank	Country	Publications	Total citations	Average citation	H-index	Total Link strength
1	USA	641	26088	40.6989	76	414
2	China	579	13952	24.0967	54	235
3	Japan	172	4676	27.186	32	88
4	Germany	161	7874	48.9068	41	202
5	Italy	94	4925	52.3936	32	123
6	Australia	82	4104	50.0488	30	105
7	Brazil	80	1957	24.4625	23	49
8	Canada	76	3606	47.4474	33	71
9	United Kingdom	73	4666	63.9178	31	134
10	France	67	4002	59.7313	24	107

### 3.3 Analysis of authors

#### 3.3.1 Contribution of authors

**Figure 4:**
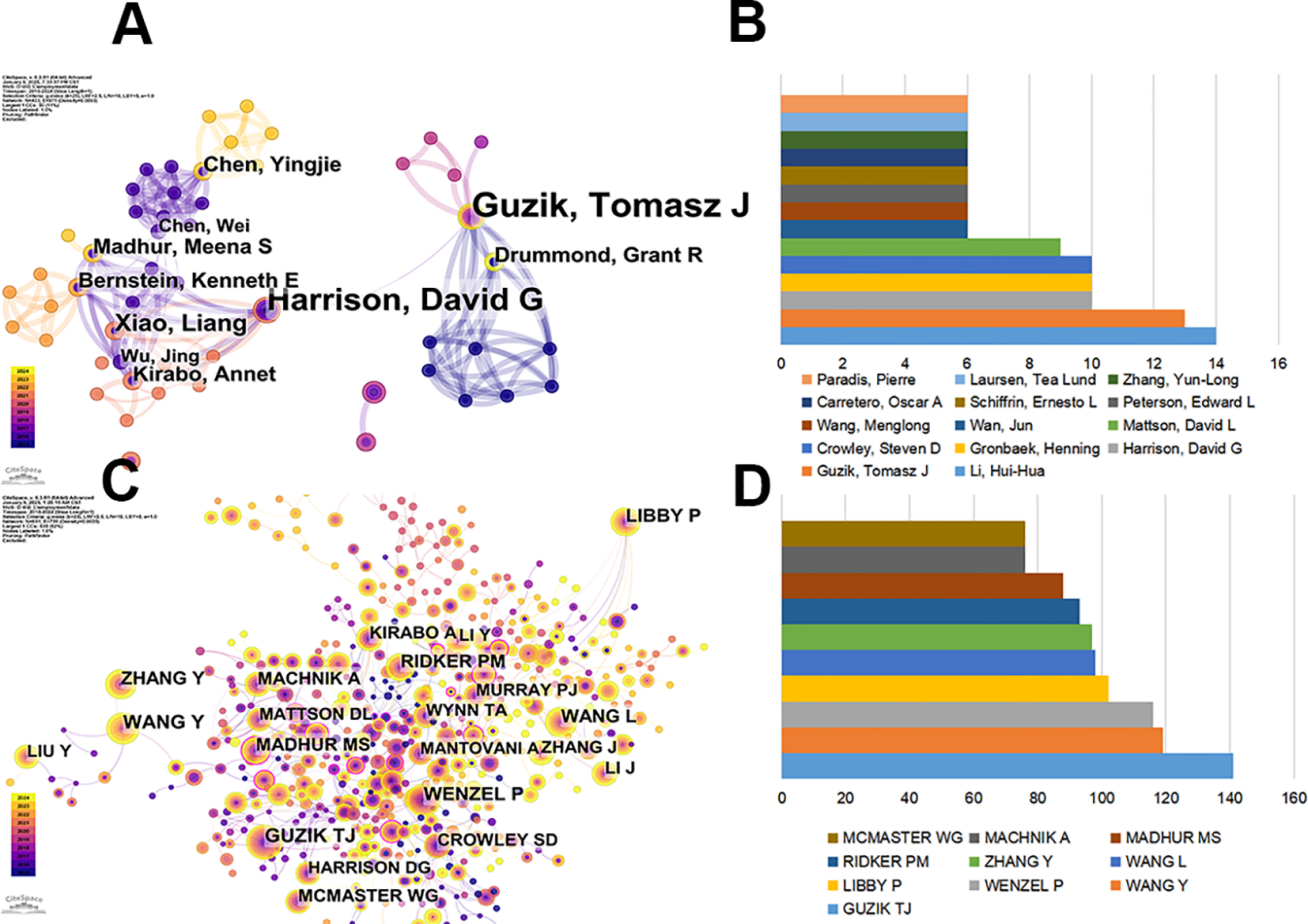
Authors contributed to the study of macrophages in hypertension. **(A)** Visualization of co-occurrence of authors based on CiteSpace. **(B)** Top 10 authors ranked by publication volume. **(C)** Co-citation visualization map of authors based on CiteSpace. **(D)** Top 10 authors ranked by co-citations.

We selected top 100.0% of most cited or frequently occurring items from each slice, with the maximum number of selected items per slice set to 1000 in Citespace. A total of 13,325 authors published articles on hypertension and macrophages from 2015 to 2024. As shown in **Table 2**, Li, Hui-Hua from Capital Medical University published the most articles (14, 12.28%), followed by Guzik, Tomasz J (13, 11.4%), Harrison, David G (10, 8.77%), Gronbaek, Henning (10, 8.77%), and Crowley, Steven D (10, 8.77%). Top 14 authors ranked by publication volume are listed in **Figure 4B**. Li, Hui-Hua (14) and Guzik, Tomasz J (13) were the top 2 authors with the most significant number of publications.

**Table 2:** Top 10 authors in terms of publication volume related to macrophages in hypertension.

Rank	Author	Institutions	Publications
1	Li, Hui-Hua	Capital Medical University	14
2	Guzik, Tomasz J	University of Edinburgh	13
3	Harrison, David G	Vanderbilt University Medical Center	10
4	Gronbaek, Henning	Aarhus University Hospital	10
5	Crowley, Steven D	Duke University	10
6	Mattson, David L	Augusta University	9
7	Wan, Jun	Wuhan University	6
8	Wang, Menglong	Renmin Hospital of Wuhan University	6
9	Peterson, Edward L	Henry Ford Health System	6
10	Schiffrin, Ernesto L	McGill University	6
11	Carretero, Oscar A	Henry Ford Hospital	6
12	Zhang, Yun-Long	Capital Medical University	6
13	Laursen, Tea Lund	Aarhus University	6
14	Paradis, Pierre	Lady Davis Institute	6

The network of relationships, presented in **Figure 4**, reflected the connections between the leading authors in the field. In the co-occurrence graph (**Figure 4A**), the parameters were set as follows: time slicing (2015–2024), years per slice (1), node type (author), selection criteria (k=25), Pathfinder, pruning sliced networks, and pruning the merged network. The size of each circle is proportional to the total number of occurrences for the author, while the thickness of the connecting lines indicates the strength of the connections between authors. We can intuitively understand the high occurrence of Guzik, Tomasz J and Harrison, David G from **Figure 4A**, where Harrison, David G is mainly concentrated before 2021, while Guzik, Tomasz J continues until 2024.

#### 3.3.2 Co-cited authors

In the co-cited graph (**Figure 4C**), the size of each circle represents the cumulative number of co-citations between authors, and the thickness of the connection indicates the strength of their relationship. A co-cited author refers to two or more authors who are cited by at least one article simultaneously, indicating that similarities in their research (23). It could be seen that GUZIK TJ, WENZEL P, WANG Y and LIBBY P had made remarkable contributions. MADHUR MS from Vanderbilt University marked by the outer purple rings on the node exhibited high centrality, indicating the role as bridges in the network. Conversely, authors displayed a low centrality (n = 0), suggesting that further exploration and collaboration are needed in this field. For evaluating the degree of connection and the influences of authors, the co-citation of authors is analyzed in **Figure 4D**. Among the top 10 authors ranked by co-citations, GUIZIK ranks first, followed by WANG Y and WENZEL P, reflecting their high impacts in related fields (**Table 3**).

**Table 3:** Top 10 authors with the highest co-citation of macrophages related to hypertension.

Rank	Author	Institutions	Count
1	GUZIK TJ	University of Edinburgh	141
2	WANG Y	Shanghai Institutes for Biological Sciences, Chinese Academy of Sciences	119
3	WENZEL P	German Centre for Cardiovascular Research	116
4	LIBBY P	Brigham and Women’s Hospital and Harvard Medical School	102
5	WANG L	Tianjin Ctr Hlth & Meteorol Multidisciplinary Inno	98
6	ZHANG Y	Shandong University	97
7	RIDKER PM	Harvard Medical School	93
8	MADHUR MS	Vanderbilt University	88
9	MACHNIK A	University of Erlangen Nuremberg	76
10	MCMASTER WG	Vanderbilt University	76

### 3.4 Contribution of active institutions

**Figure 5:**
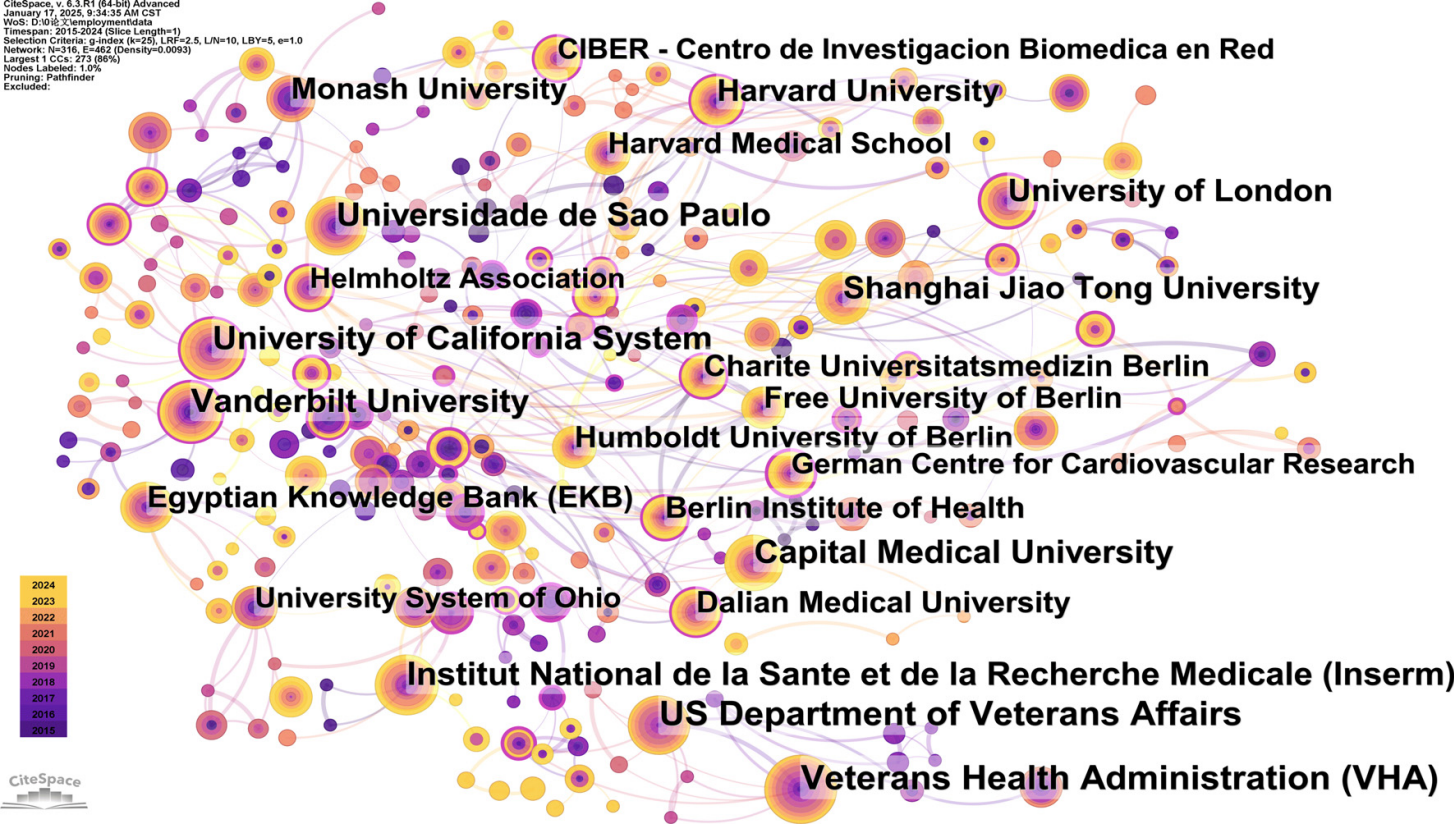
Network of institutions conducting research related to macrophages in hypertension.

The top 10 universities with the highest number of publications and centrality are listed separately in **Table 4** (due to parallel reasons, the centrality ranking is in the top 12). Only the University of London is among the top ten institutions in terms of both publication quantity and centrality. In the top 10 institutions ranked by count, the US Department of Veterans Affairs was led with the highest number of publications (50), followed by Veterans Health Administration (49) and University of California System (43). University of London ranked eighth, while its centrality ranked third. In terms of centrality, National Institutes of Health - USA (0.38) and Pennsylvania Commonwealth System of Higher Education (0.32) are the top two in institutions. Institutional co-occurrence network analysis, conducted by CiteSpace (**Figure 5**), aimed to identify organizations or institutions with relatively mature research. In the figure, the nodes represent institutions, with their size proportional to the volume of publications. The connections between nodes indicate the cooperative relationships among institutions. The color of the connection represents the start time of these collaborations, while the thickness reflects the strength of the relationship. Specifically, US Department of Veterans Affairs, Harvard University, Vanderbilt University, University of California System and University of London were marked by the purple circles outside, representing the potential leaders of significant breakthroughs. Overall, data and graphs demonstrate that cooperation between leading institutions needs to be strengthened.

**Table 4:** Top 10 institutions performing related studies ranked by count and centrality.

Rank	Institutions	Count	Rank	Institutions	Centrality
1	US Department of Veterans Affairs	50	1	National Institutes of Health (NIH) - USA	0.38
2	Veterans Health Administration (VHA)	49	2	Pennsylvania Commonwealth System of Higher Education (PCSHE)	0.32
3	University of California System	43	3	University of London	0.28
4	Institut National de la Sante et de la Recherche Medicale (Inserm)	43	4	Max Delbruck Center for Molecular Medicine	0.26
5	Vanderbilt University	42	5	German Centre for Cardiovascular Research	0.24
6	Universidade de Sao Paulo	42	6	University of Texas System	0.24
7	Capital Medical University	40	7	Vrije Universiteit Amsterdam	0.24
8	University of London	35	8	Imperial College London	0.23
9	Shanghai Jiao Tong University	35	9	University of Texas Health Science Center Houston	0.23
10	Harvard University	33	10	Medical University of Vienna	0.21
			11	Baylor College of Medicine	0.21
			12	Mayo Clinic	0.21
			13	Johns Hopkins University	0.21

### 3.5 Contribution of journals

**Figure 6:**
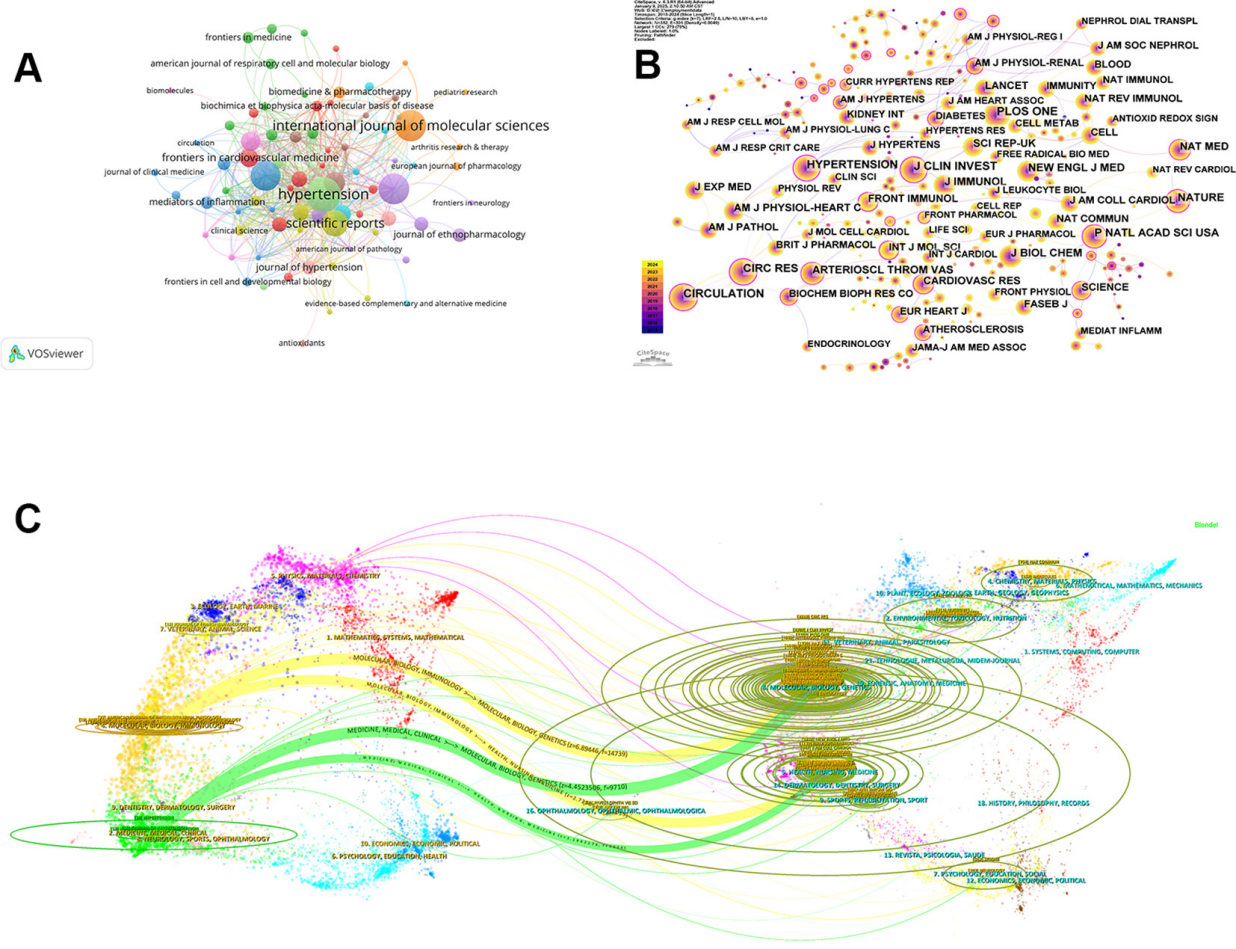
Visualization of journals related to macrophages and hypertension. **(A)** Network visualization of source journals based on VOSviewer. **(B)** Visualization of cited journals using CiteSpace. **(C)** Analysis of the dual-map overlay.

A total of 351 journals were involved in the publication of articles related to hypertension and macrophages. Among the 10 journals with the highest number of co-citations and centrality listed in **Table 5**, 70% were US-based, with the remaining from UK. HYPERTENSION (0.89) had the highest centrality among the journals listed in **Table 5**, followed by CIRC RES (0.77) and CIRCULATION (0.39), where there are also relatively abundant quantities on co-citation. Similarly, Nature had a relatively high centrality (0.36), although the number of co-citation (798) ranked seventh, reflecting the relatively higher level of publication and innovation capabilities. On the contrary, PLOS ONE had the highest co-citation (1047), but its centrality did not rank in the top ten. **Figures 6A, B** depicted the links and the number of indexed journals. The journals primarily focused on nutrition, cardiovascular, and molecular biology, suggesting that researchers in related fields may prioritize them for their submissions. Both the top 10 journals with the largest number of publications and those with the highest centrality were classified as the JCR partitions of Q1 or Q2 (representing the top 25% and 25–50% of Impact Factor distribution), indicating the reliable quality of the documents included from WoS. The dual-map overlay of journals showed the distribution of relationships among journals, highlighting the key connections and signaling multidisciplinary cross-cooperative studies. The citing journals are displayed on the left, while the cited journals are on the right, with the colored paths reflecting the citation relationships. **Figure 6C** showcases 4 significant citation pathways. The two yellow lines represent that research published in Molecular/Biology/Genetics journals and Health/Nursing/Medicine journals has been cited in Molecular/Biology/Immunology journals, while the two green lines show that studies from Molecular/Biology/Genetics journals and Health/Nursing/Medicine journals have also been cited in Medicine/Medical/Clinical journals (14). This analysis provides valuable insights for comprehensively understanding the research landscape related to the connection between macrophages and hypertension.

**Table 5:** Top 10 journals in terms of co-citation related to macrophages and hypertension.

Rank	Journal	Count	JCR Partitions	Rank	Journal	Centrality	JCR Partitions
1	Plos One	1047	Q2	1	Hypertension	0.89	Q1
2	Circulation	1033	Q1	2	Circulation Research	0.77	Q1
3	Journal of Clinical Investigation	1029	Q1	3	Circulation	0.39	Q1
4	Circulation Research	947	Q1	4	Journal of Clinical Investigation	0.37	Q1
5	Hypertension	932	Q1	5	Nature	0.36	Q1
6	Proceedings of the National Academy of Sciences of the United States of America	853	Q1	6	Diabetes	0.36	Q1
7	Nature	798	Q1	7	Diabetologia	0.36	Q1
8	Journal of Biological Chemistry	747	Q2	8	American Journal of Physiology-renal Physiology	0.35	Q1
9	Arteriosclerosis Thrombosis and Vascular Biology	738	Q1	9	Journal of Clinical Endocrinology & Metabolism	0.3	Q1
10	New England Journal of Medicine	710	Q1	10	Frontiers in Immunology	0.28	Q1

### 3.6 Analysis of references

#### 3.6.1 Co-citation of references

**Figure 7:**
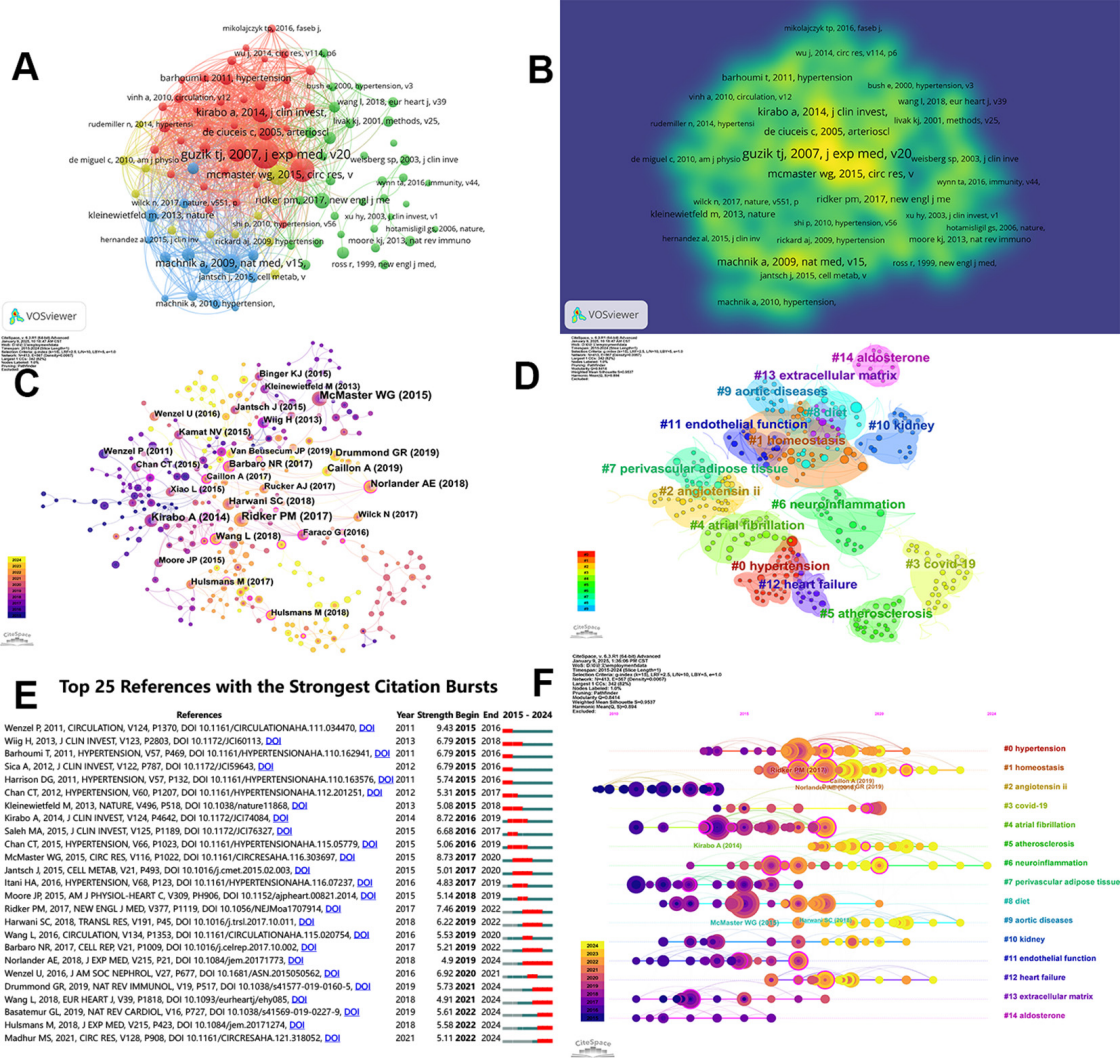
Visualization of co-cited literature on macrophages related to hypertension. **(A)** VOSviewer based references co-citation network. **(B)** The density visualization of co-cited references based on VOSviewer. **(C)** References co-citation network based on CiteSpace. **(D)** CiteSpace based clustering analysis of co-cited references. **(E)** Top 25 references in terms of citation bursts. **(F)** A timeline of the 15 largest clusters.

Co-citation analysis is a key aspect of reference analysis, revealing the strength of the connections between references. VOSviewer identified the top 5 most co-cited references, and citespace was used for further analysis of these the co-cited references. In **Figure 7A**, different colors represent the citation directions of various references, with the lines between them indicating the strength of their citation network relationships. **Figure 7B** presents a more concise and clear visualization of their density visualization.

#### 3.6.2 Clusters of references

In addition, as shown in **Figure 7C**, the parameters were set as follows: time slicing (2015–2024), years per slice (1), node type (cited reference), selection criteria (k=15), Pathfinder, and Pruning sliced networks. A co-occurrence network was obtained from Citespace, consisting of 413 nodes, 567 edges, and a density of 0.0067. McMaster WG, Kirabo A, and Ridker PM had larger nodes, reflecting the interest and recognition of their research in related fields. After filtering out 15 clusters with low silhouette scores, we identified four key aspects, as shown in **Figure 7D**. The Modularity Q was 0.8414, and the Mean Silhouette was 0.9537, indicating the clusters are well-defined and the framework is clear. To gain insight into the development of clusters over the last decade, a reference co-citation time-view map was constructed, as shown in **Figure 7F**. Hot-spot references from each period were selected based on the citation frequency and centricity. **Figure 7E** shows the top 25 references with the strongest citation bursts, indicating shifts in study focus. Among them, the strongest citation burst was for the 2011 article “Lysozyme M-Positive Monocytes Mediate Angiotensin II-Induced Arterial Hypertension and Vascular Dysfunction”. This article highlighted the critical role of infiltrating monocytes with a proinflammatory phenotype and macrophages in ATII-induced vascular dysfunction and arterial hypertension (24). According to **Table 6**, the most cited article was authored by Guzik TJ, who identified a previously undefined role for T cells in the genesis of hypertension, supporting the involvement of inflammation in this widespread disease. He supposed that T cells might represent a novel therapeutic target for the treatment of high blood pressure (25). Additionally, Machnik A (2009) concluded through experimental findings that TonEBP-VEGF-C signaling in MPS cells serves as a crucial factor for regulating extracellular volume and maintaining blood pressure equilibrium, identifying VEGFC as an osmosensitive gene driven by hypertonicity that plays a key role in salt-induced hypertension (26). Furthermore, IL-17 was found to be essential for sustaining angiotensin II-induced hypertension and vascular dysfunction, providing a possible therapeutic target (27). McMaster WG (2015) examined the relationship between oxidative stress and immune response in hypertension, indicating that both innate and adaptive immune system cells contribute to damage and dysfunction in end organs associated with hypertension, where attenuating the activation of these cells could present a potential treatment approach (28).

**Table 6:** Top 5 references with the most co-citation associated with macrophages related to hypertension.

Rank	First author	Journal	Year	Citations	TLS
1	Tomasz J Guzik	j exp med, v204, p2449 (25)	2007	121	1245
2	Philip Wenzel	circulation, v124, p1370 (24)	2011	99	1108
3	Meena S Madhur	hypertension, v55, p500 (27)	2010	77	1038
4	William G McMaster	circ res, v116, p1022 (28)	2015	77	685
5	Agnes Machnik	nat med, v15, p545 (26)	2009	75	883

### 3.7 Keyword analysis

#### 3.7.1 Cluster of keyword

**Figure 8:**
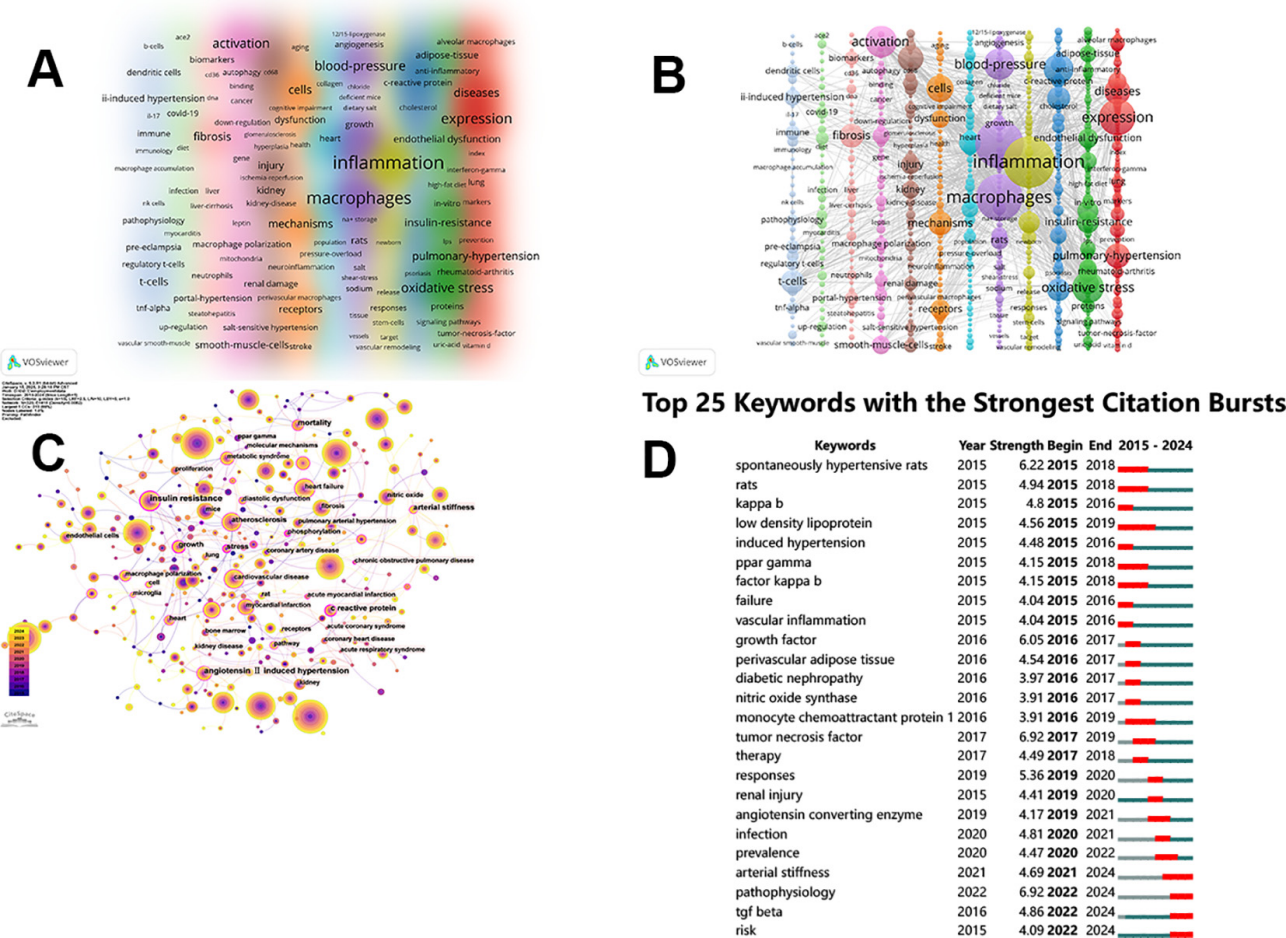
Keyword map associated with macrophages research related to hypertension. **(A)** The keywords clusters. **(B)** Density visualization of keywords clusters. **(C)** Keyword visualization based on CiteSpace. **(D)** Top 25 keywords with the strongest citation bursts.

Keywords represent the core concepts of an article, summarizing its research focus or theme. Therefore, conducting an in-depth examination of keywords can illuminate important changes in research trends within a specific discipline. Through the cluster analysis (**Figure 8A**) performed on VOSviewer, 12 distinct clusters have been identified, each represented by different colors, revealing various research directions. The minimum number of keyword occurrences was set at 5, out of the 8712 keywords, with a total of 807 meeting this threshold. The largest group is Cluster 1 (red), containing 100 keywords, including alveolar macrophages, diseases, expression, dysfunction, rheumatoid-arthritis, tumor-associated macrophages, etc. Cluster 2 (green) follows, comprising 84 keywords, including adipose-tissue, endothelial dysfunction, insulin-resistance, oxidative stress, proteins, signaling pathways, etc. Cluster 3 (blue) has 77 keywords that include c-reactive protein, cholesterol, psoriasis, and so forth. Cluster 4 (yellow) consists of 64 keywords, largely focused on inflammation, newborn, release, responses, stem-cells, target and vascular remodeling. Cluster 5 (purple) is made up of 60 keywords, which encompasses angiogenesis, blood-pressure, growth, macrophages, rats, sodium tissue, and others. Cluster 6 (light blue) contains 58 keywords, featuring collagen, heart and population. Cluster 7 (orange) has 52 keywords, which consist of aging, cells, dysfunction, mechanisms, receptors, etc. Cluster 8 (brown) includes 51 keywords, mainly including injury, kidney, polarization and salt-sensitive hypertension. Cluster 9 (pink) comprises 44 keywords, featuring autophagy, cancer, binding, smooth-muscle-cells, macrophage polarization, mitochondria, down-regulation and activation. Cluster 10 consists of 41 keywords, with included biomarkers, cd36, fibrosis, liver, neutrophils, portal-hypertension and steatohepatitis among them. Cluster 11 (light green) contains 37 keywords, such as ace2, covid-19, diet, inflection, myocarditis, up-regulation, and Cluster 12 totals 36 keywords, which includes b-cells, dendritic cells, ii-induced hypertension, immune, macrophage accumulation, nk cells, pathophysiology, pre-eclampsia, t-cells TNF-alpha and others. **Table 7** showed the top 10 keywords based on frequency. Inflammation (668 occurrences) appeared most frequently, with a high Total Link Strength (TLS) of 5269, representing the main and central keyword in macrophage-related hypertension research.

**Table 7:** Top 10 keywords related to macrophages and hypertension.

Rank	Keywords	Occurrences	TLS
1	Inflammation	668	5269
2	Hypertension	596	4638
3	Macrophages	554	4281
4	Expression	333	2505
5	Activation	258	2082
6	Oxidative Stress	256	2104
7	Blood-Pressure	251	2074
8	Atherosclerosis	231	1746
9	Cells	183	1370
10	Diseases	175	1252

#### 3.7.2 Co-occurrence and strongest citation bursts of keyword

We conducted a keyword analysis in Citespace (**Figure 8C**) to determine the distribution of keywords over time. Among them, angiotensin II induced hypertension, heart failure, growth, annual stiffness, and atherosclerosis were marked with purple circles, which also verify the hotspots of some keywords in **Table 7** and **Figure 8D**. Among the top 25 keywords with the strongest citation bursts (**Figure 8D**), we focused on those with significant research implications. The top five keywords exhibiting the strongest burst intensity were pathophysiology (6.92), tumor necrosis factor (6.92), spontaneously hypertensive rats (6.22), growth factor (6.05), and responses (5.36). The keyword that maintained a burst for the longest time was low-density lipoprotein (2015-2019). In recent years, keywords like arterial stiffness, pathophysiology, TGF beta, and risk have surfaced as key topics of study.

## 4 Discussion

### 4.1 General information

To comprehensively summarize and visually analyze research trends and hotspots in hypertension related to macrophages, we used Microsoft Excel 2021, VOSviewer 1.6.20, CiteSpace 6.3.R1 and Scimago Graphica 1.0.46. The bibliometric analysis was conducted to 2013 original articles and reviews published between 2015 and 2024 in WoS. The results revealed a steady increase in both the annual number of publications and citations over the past decade, indicating a clear and upward trend. This surge of interest highlights the growing importance of research on macrophages related to hypertension. Among them, the number of annual publications in 2021 is the highest in the decade, and the annual citation volume is also approaching its peak in 2021, 2022 and 2023, except for 2024, reflecting a more prosperous exploration and collaboration on hypertension and macrophages since 2021.

These 2,013 English-language papers published in 351 journals from 2015 to 2024 by 315 universities/institutions in 83 countries/regions. Notably, the United States positioned itself at the heart of this research field, leading in scientific study and influencing the research directions. The centralization of contributions from key countries reflects the widespread cooperation among countries in this research area.

### 4.2 Important contributing authors and journals

Li, Hui-Hua from Capital Medical University with the most publications, and GUZIK TJ from the University of Edinburgh with the most co-citations, are major contributors in this field. Li, Hui-Hua’s highly cited paper posited that Angiotensin II-induced infiltration of monocytes in the heart is predominantly mediated by CXCL1-CXCR2 signaling, which plays a crucial role in initiating and exacerbating cardiac remodeling. This study concluded that the inhibition of CXCL1 and/or CXCR2 might be a promising therapeutic approach for treating hypertensive heart diseases (29). While Li, Hui-Hua concentrates on the pathophysiology, GUZIK TJ focus on the clinical associations in recent 10 years, such as Stable Cardiovascular Disease (30), COVID-19 (31) and Diabetes (32). Besides, *HYPERTENSION* and *PLOS ONE* are highlighted as the primary journals in this field, offering valuable resources for researchers seeking to access or publish groundbreaking work. *Circulation*, *Circulation Research*, *Hypertension*, *Arteriosclerosis Thrombosis and Vascular Biology*, *Journal of Clinical Investigation*, and *Nature* are more likely to have significant or even revolutionary impacts on related fields, the metrics of which can also provide further insights for scholars.

### 4.3 Hotspots and frontiers

Co-cited references analysis is instrumental in identifying research trends, identifying primary clusters in the study of hypertension associated with macrophages (**Figure 7D**). In addition to the lifestyle factor of #8 diet, it can be mainly divided into two clusters: the regulatory mechanisms of hypertension and diseases related to hypertension. The regulatory mechanisms of hypertension include #1 homeostasis, #2 angiotensin ii, #6 neuroinflammation, #7 perivascular adipose tissue, #10 kidney, #11 endothelial function, #13 extracellular matrix, #14 aldosterone. Diseases related to hypertension include #0 hypertension, #3 covid-19, #4 atrial fibrillation, #5 atherosclerosis, #9 aortic diseases, #12 heart failure. Co-cited references are regarded as essential to the research within a specific domain, while the progression of keywords can indicate the advancement of hot-spots. Both co-cited references and keywords are instrumental in shaping the direction of future investigations in this area (33). WENZEL U proposed to support immunity as a regulatory factor for blood pressure and hypothesized the mechanism of inflammation and hypertension (34). Although the duration was only one year, the explosive intensity ranked among the top five, reflecting the attention of the relevant field. Annet Kirabo utilized mouse models characterized by elevated vascular production of reactive oxygen species (ROS) to establish a pathway that connects vascular oxidative stress to immune activation and aortic stiffness. This research offers valuable insights into the systemic inflammation associated with prevalent vascular diseases (35). The duration of the burst reached three years, with a strength of 8.73. Similarly, **Table 7** also lists inflammation and oxidative stress, with inflammation being the highest occurrence (669) and TLS being the highest (5269). In the analysis performed in this research, relevant topics emerged in both the clustering of co-cited references and the groupings of keywords, suggesting that these subjects represent significant areas of investigation within this discipline (**Figure 8A**). The clustering of keywords and analysis of citations helps us clearly summarize emerging research trends. The 12 clusters provide insights into the current areas of interest and potential future research directions in this discipline. In the past decade, the keywords with strong citation bursts have primarily concentrated in pathophysiology, spontaneously hypertensive rats and growth factor, showing the potentiality for deeper exploration in related area. Until 2024, the attention to pathophysiology is still ongoing and the intensity is as high as 6.92. Sun, Yi’s team has demonstrated through animal experiments that TWIST1 in macrophages plays a critical role in mediating foam cell formation and increasing the vulnerability of atherosclerotic plaque during hypertension. It points out that targeting TWIST1 may offer a promising new therapeutic approach for slowing the progression of atherosclerosis in hypertension (36).

#### 4.3.1 Inflammation

Inflammation is a nonspecific defense mechanism of the body, playing a critical role in adaptive remodeling in response to injury (37). Hypertension is characterized by a chronic inflammatory state, marked by excessive macrophage activation and M1 polarization, along with the production of proinflammatory cytokines and growth factors by activated innate immune cells and endothelial cells (38). While chronic inflammation has been established as a contributing factor to the pathogenesis of hypertension, the complex molecular mechanisms linking inflammation and immunity to affect elevated blood pressure remain largely theoretical (4).

Inflammation is of paramount importance in the development process of hypertension (39). The low degree inflammatory diseases of hypertension usually have no obvious symptoms, except for elevated levels of inflammatory biomarkers (40). In the pathophysiological mechanism of hypertension, macrophage colony-stimulating factor (m-CSF), a monocyte chemokine, regulates the inflammatory response by modulating the effector functions of mature monocytes and macrophages, as well as stimulates the production of other cytokines, adhesion molecules, and growth factors (41). In addition, activation of the RAS system in the body can stimulate macrophages to secrete various inflammatory factors (42). TNF α could activate the NF κ B signaling pathway and NADPH oxidase activity in endothelial cells to increase the generation of ROS, which cause the result of the inhibition of endothelial cell synthesis of NO and dysfunction of endothelial cell (43). Consistently, endothelial cells are also targets of IL-1β and can produce IL-1β (44). Several scholars have assessed the regulatory role of macrophages in blood pressure using genetic and pharmacological approaches in different animal models. A plethora of animal studies across various models (39, 45, 46) demonstrate that modulating inflammatory responses and the release of effector cytokines may help reduce blood pressure elevations and mitigate the progression of vascular, cardiac, and renal injuries. Moreover, small molecules or neutralizing antibodies directed at immune mediators have been employed to assess the effects of specific blockade on induced hypertension (47). These interventions have focused on both the innate (e.g., IL-1, TLR4) and adaptive (e.g., IL-17, CD80/86) components of the immune system. A thorough examination of these studies aids in identifying promising therapeutic targets while also emphasizing the influence of treatment protocols and the choice of animal models on blood pressure and target organ damage outcomes (47).

As of now, there is no clear evidence that the conventional use of anti-inflammatory agents effectively treats hypertension. Current experiments indicate that Minocycline (48), Mycophenolate mofetil (49) and long-term use of immunosuppressive drugs (50) have a significant impact on lowering blood pressure. Additionally, it has been substantiated that statins also contribute to antihypertensive effects. The observed blood pressure reduction in patients treated with statins is clinically significant and only partially associated with the lipid-lowering effect (51). The potential mechanism may involve the protective role of statins on vascular endothelium, including inhibiting the production of reactive oxygen species (ROS), reducing the circulating levels of pro-inflammatory cytokines, and inhibiting the expression of adhesion molecules on vascular endothelial cells and smooth muscle cells (4). The current research focus on inflammation could deepen conventional understanding of the pathophysiological frameworks in hypertension. Investigating macrophage metabolism in disease states may offer promising therapeutic strategies for hypertension. While, in metabolic syndrome, multiple mechanisms may mediate M1 polarization, substantial evidence suggests that alterations in macrophage metabolism—driven by changes in metabolic substrates and pro-inflammatory lipids—play an important role, so evaluating strategies to improve macrophage metabolism is essential for advancing hypertension treatment options (52). While inflammation especially macrophage is a prospective target, further development of anti-inflammatory and immunomodulatory therapies is necessary (53). Following the endorsement of renin inhibitors and the current clinical trials of baxdrostat for managing hypertension (54), it is crucial to examine how these innovative treatments may influence the inflammatory pathways associated with high blood pressure (47).

#### 4.3.2 Oxidative stress

Oxidative stress plays a crucial role in the initiation and progression of hypertension and is considered a potential common etiological factor in various cardiovascular diseases. Oxidative stress is linked to complications such as the development and worsening of vascular dysfunction and target-organ damage. A chronic inflammatory state, driven by the excessive production of superoxide anions (·O2) and hydrogen peroxide (H2O2) by endothelial cells, monocytes, and macrophages, exacerbates oxidative stress, resulting in vascular dysfunction and target-organ damage in the course of hypertension (55). The production of reactive oxygen species (ROS), including superoxide anions and hydrogen peroxide, by these cells contributes to oxidative stress and induces a low-grade inflammatory environment. Chronic inflammation and oxidative stress mutually reinforce each other by activating transcription factors such as nuclear factor (NF)-κB. Additionally, superoxide can diminish nitric oxide (NO) availability by reacting with NO to form peroxynitrite. The cofactor 4-tetrahydrobiopterin (BH4), involved in endothelial nitric oxide synthase (eNOS) function, can be disrupted by peroxynitrite oxidation, further enhancing oxidative stress (56). Evidence suggests that substantial ROS production by activated macrophages can lead to irreversible damage to vascular endothelial cells and microvascular remodeling, increasing peripheral arteriolar resistance (57). Persistent inflammation elevates ROS levels, which in turn causes oxidative stress and endothelial dysfunction. The regulation of oxidative stress is critical in controlling macrophage function, though the specific signaling mechanisms involved are not fully understood. Gaining insight into the molecular mechanisms underlying the metabolic regulation of macrophage inflammatory processes could offer new strategies for targeting immune metabolism and inflammation.

## 5 Conclusion

This research provides a comprehensive bibliometric analysis of hypertension and macrophages from 2015 to 2024, offering valuable insights for potential collaborations between researchers and institutions. The interaction mechanisms between macrophages and hypertension remain uncertain and require further exploration. Furthermore, macrophages may indirectly influence the development of hypertension by affecting the progression of specific diseases. In summary, macrophages show potential as a target for the treatment of hypertension, further research is essential to fully understand their role and efficacy. The reduction of cardiovascular risk through immune modulation occurs not solely through lowering blood pressure but also through more extensive mechanisms like oxidative stress, endothelial function, vascular remodeling, and hormonal regulation, so relying solely on systemic immunosuppressive approaches may not be the most effective strategy for controlling hypertension, and a combination of other methods may be necessary. Furthermore, there is a notable shortage of clinical data, and investigating the effects of targeted immune therapies on human hypertension could represent a promising avenue for future research in clinical.

## Data Availability

The original contributions presented in the study are included in the article/**Supplementary Material**. Further inquiries can be directed to the corresponding author/s.
