# Supplementary material for: Global trends and hotspots in macrophage research related to hypertension from 2015-2024: bibliometric research and visualization analysis
Source: Front Immunol. 2025 Mar 5;16:1501432. doi: 10.3389/fimmu.2025.1501432 (PMC11920128; doi:10.3389/fimmu.2025.1501432)
Supplement: Supplementary file 1 [file Table1.docx]

Supplementary Material

Supplementary Information: Search Strategy Implementation

Supplementary table 1

| Rank | Search phrases |
| --- | --- |
| #1 | TS=("Macrophages" OR "Macrophage*" OR "Macrophagy" OR "Macrophagic" OR "Macrophagocyte*") |
| #2 | TS=("Hypertension*" OR "High Blood Pressure*" OR "Hypertensive*" OR "Hyperpiesia*" OR "Hyperpiesis") |
| #3 | TI=("Pulmonary Hypertension*" OR "Pulmonary Arterial Hypertension*" OR "Ocular Hypertension*" OR "Hypertensive Retinopath*" OR "Intracranial Hypertension*" OR "Portal Hypertension*") |
| #4 | #1 AND #2 NOT #3 |

The data for Figure 2 was directly exported from Web of Science, as shown in the table below, and can be visualized using a bar chart in Excel.

Supplementary table2

|  | Annual Publications | Cumulative Publications | Annual Citations | Cumulative Citations |
| --- | --- | --- | --- | --- |
| 2015 | 167 | 167 | 187 | 187 |
| 2016 | 183 | 350 | 930 | 1117 |
| 2017 | 166 | 516 | 1957 | 3074 |
| 2018 | 191 | 707 | 3060 | 6134 |
| 2019 | 196 | 903 | 4469 | 10603 |
| 2020 | 234 | 1137 | 6961 | 17564 |
| 2021 | 246 | 1383 | 10084 | 27648 |
| 2022 | 218 | 1601 | 10310 | 37958 |
| 2023 | 205 | 1806 | 10238 | 48196 |
| 2024 | 207 | 2013 | 11316 | 59512 |

Figure 3A and Figure 3B were created using VOSviewer (version 1.6.20), with specific parameters provided in Supplementary Figure 1A and 1B. Among them, the first letter is replaced with capital letters. Figure 3C was created using Scimago Graphica (1.0.46), with specific parameters provided in Supplementary Figure 1C-1F. Figure 3D was created using Scimago Graphica (1.0.46), with specific parameters provided in Supplementary Figure 1G-1J. Figure 3E (Top 10 countries with the highest H-index) and Figure 3F (Top 10 most frequently co-cited countries) were generated directly from bar charts in EXCEL, without the need for additional parameters.


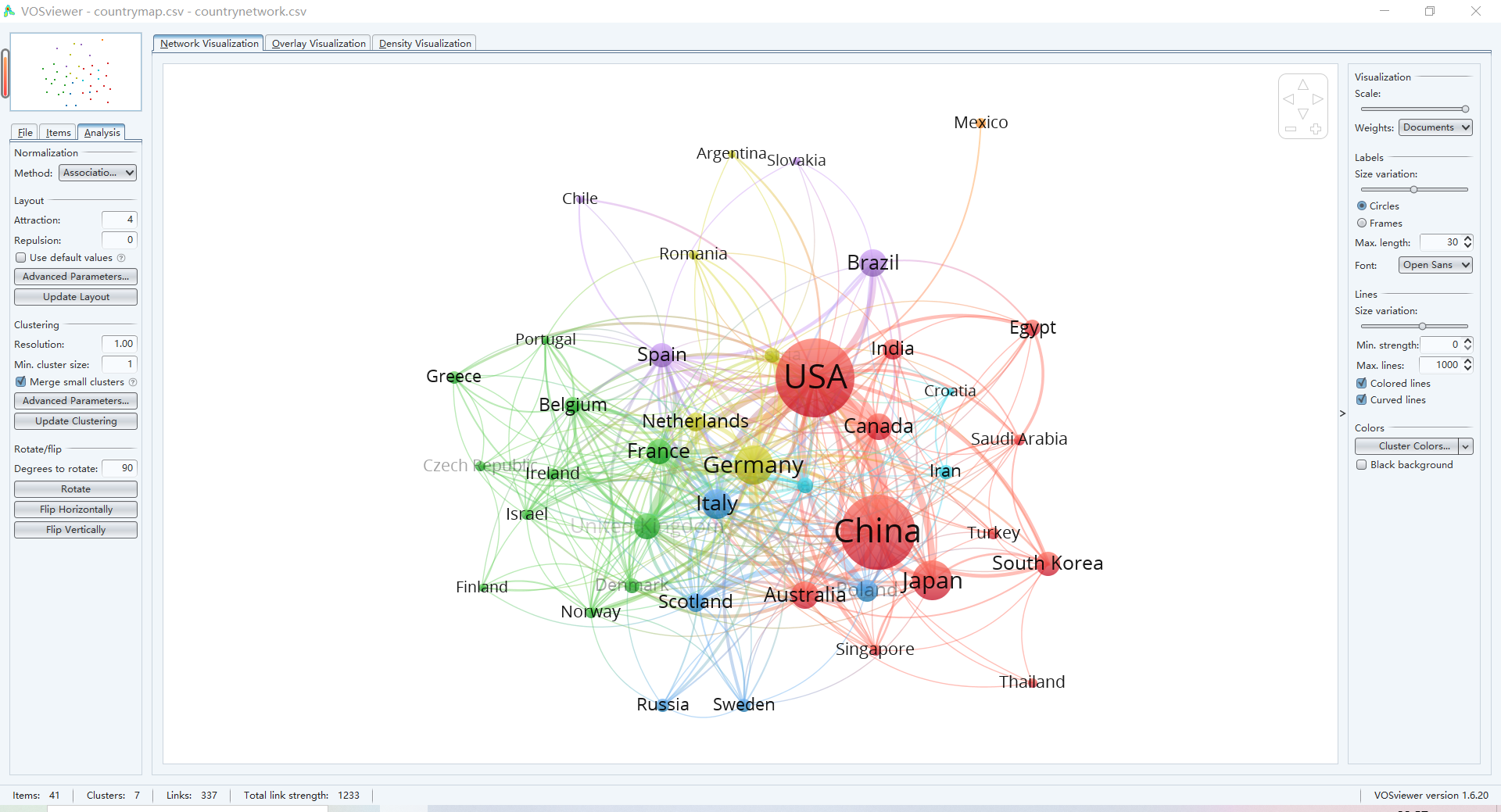


Supplementary Figure 1A：Overall parameters for collaborative network map.


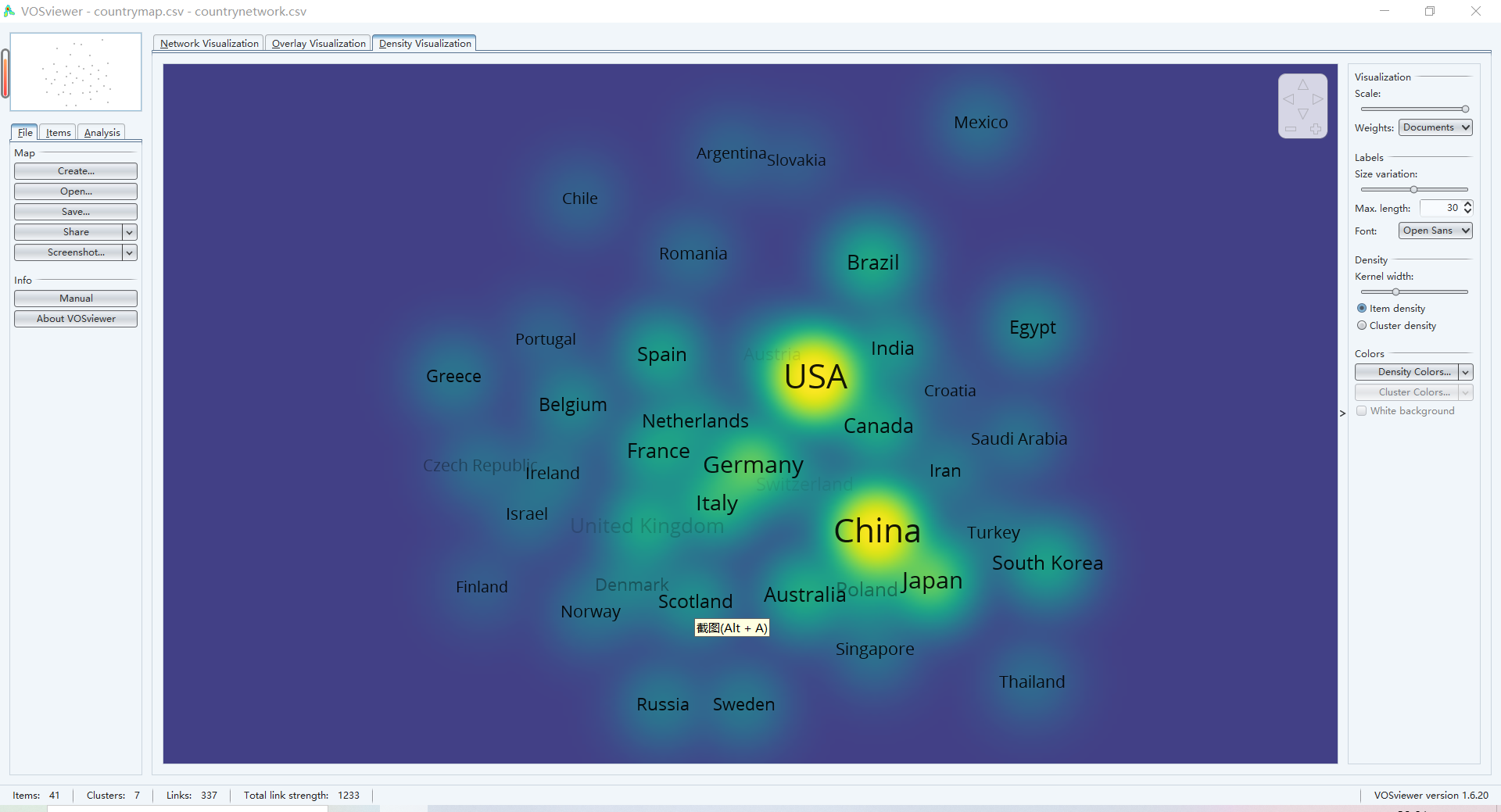


Supplementary Figure 1B：Overall parameters for Visualization of the participation density of all countries and regions


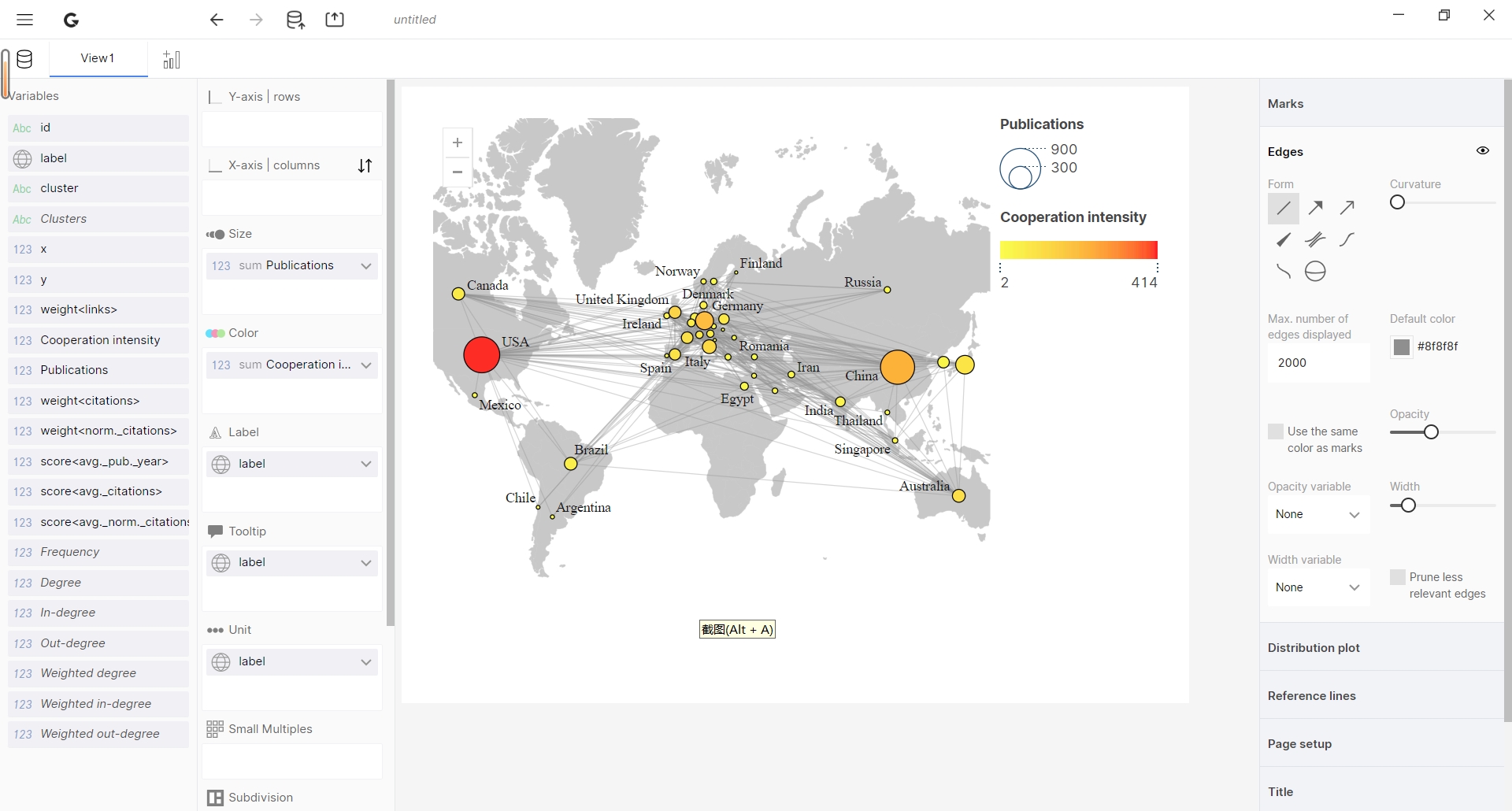
Supplementary Figure 1C：Overall parameters for global geographical distribution and network visualization


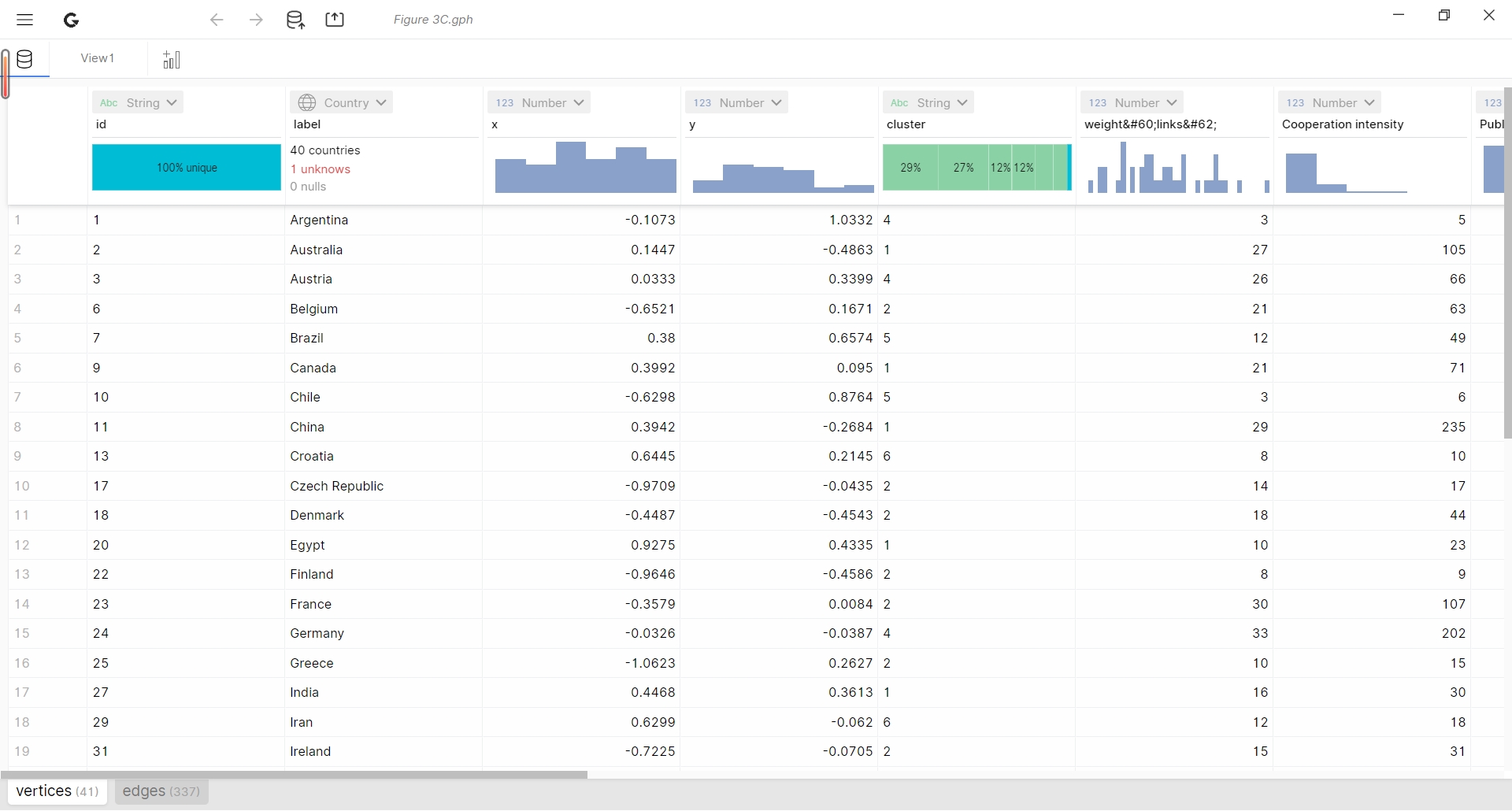
Supplementary Figure 1D：Co-networks parameters 1 for global geographical distribution and network visualization


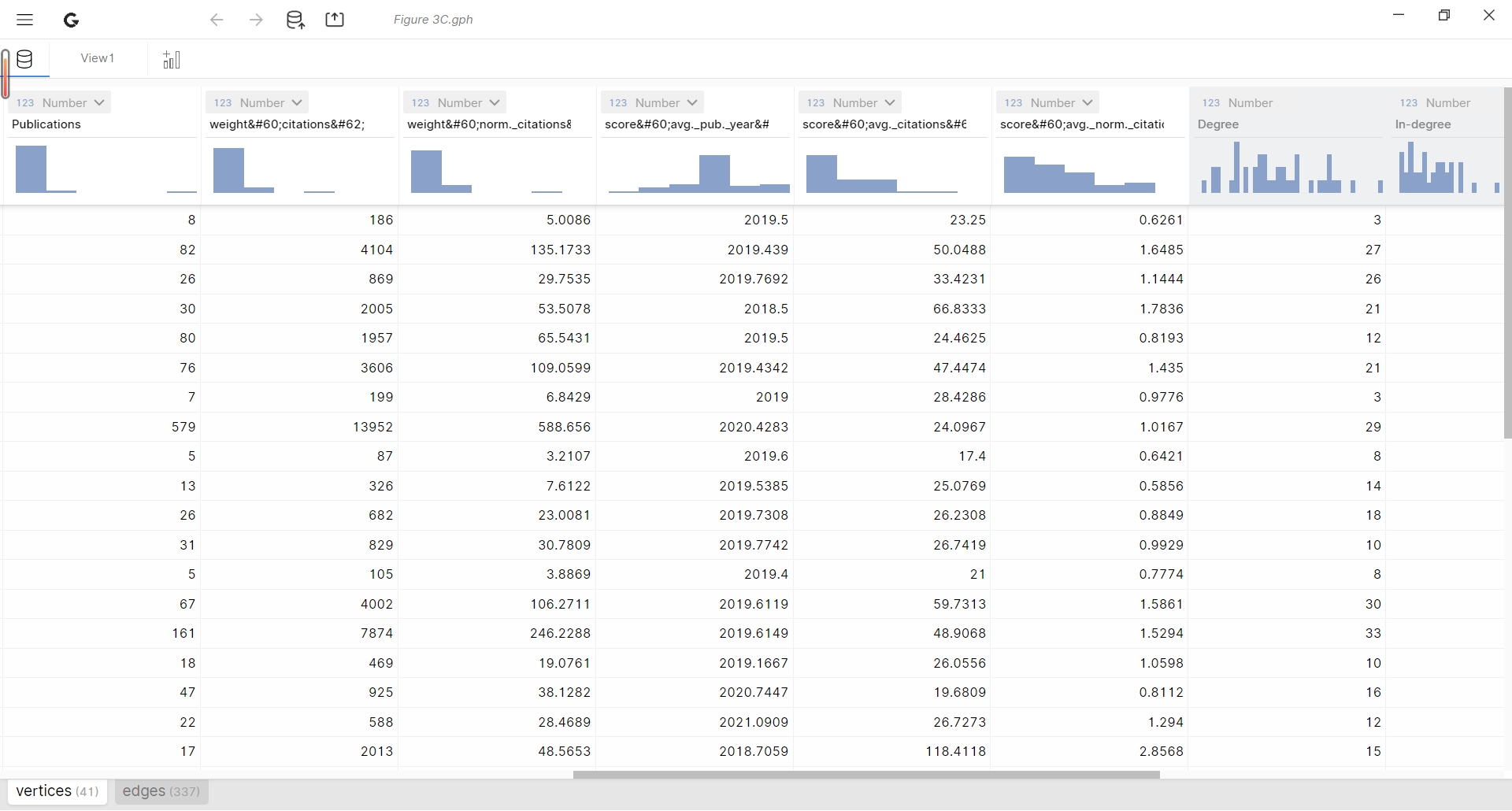
Supplementary Figure 1E：Co-networks parameters 2 for global geographical distribution and network visualization


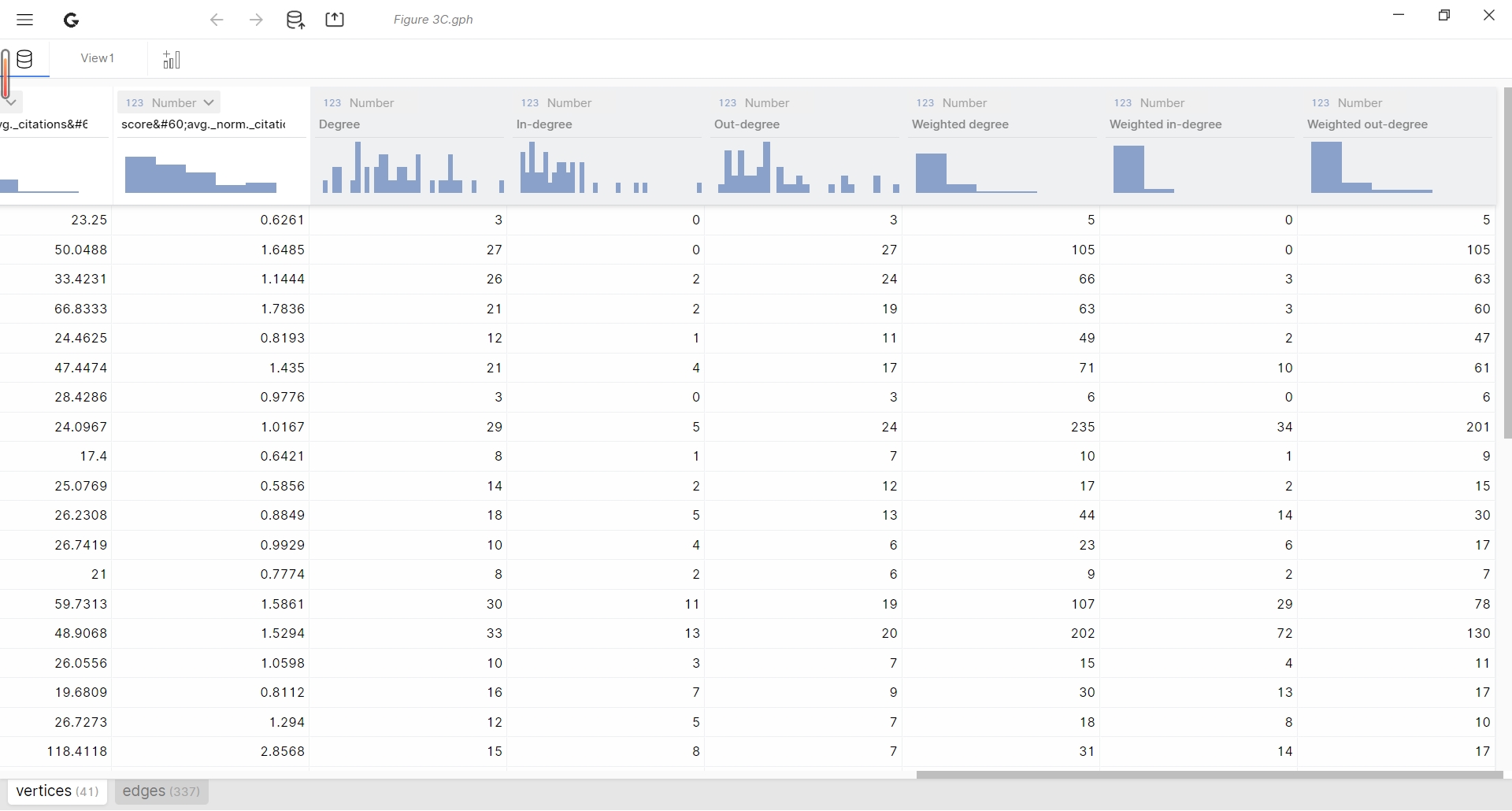


Supplementary Figure 1F：Co-networks parameters 3 for global geographical distribution and network visualization


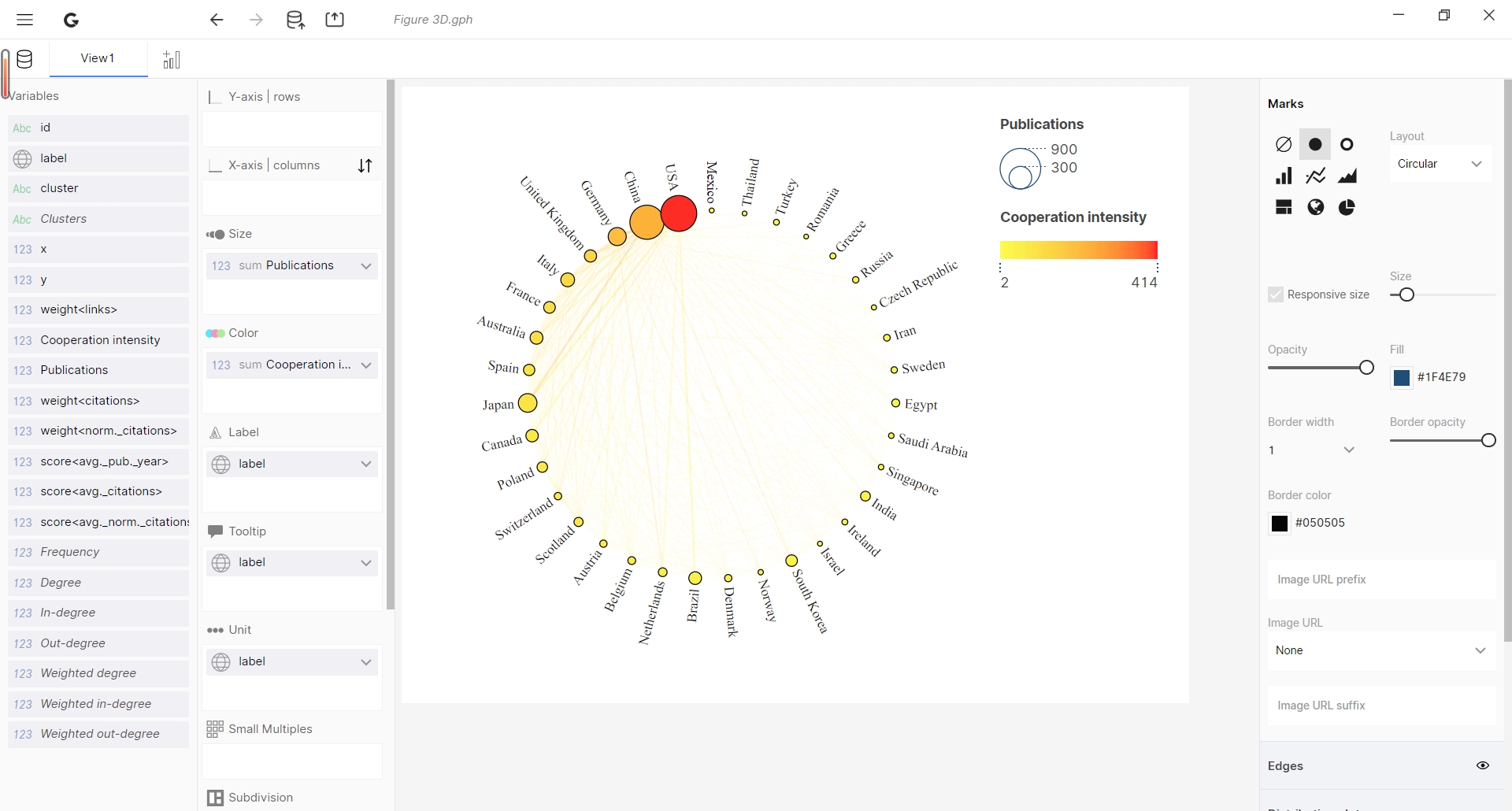


Supplementary Figure 1G：Overall parameters for Top 30 countries visualization


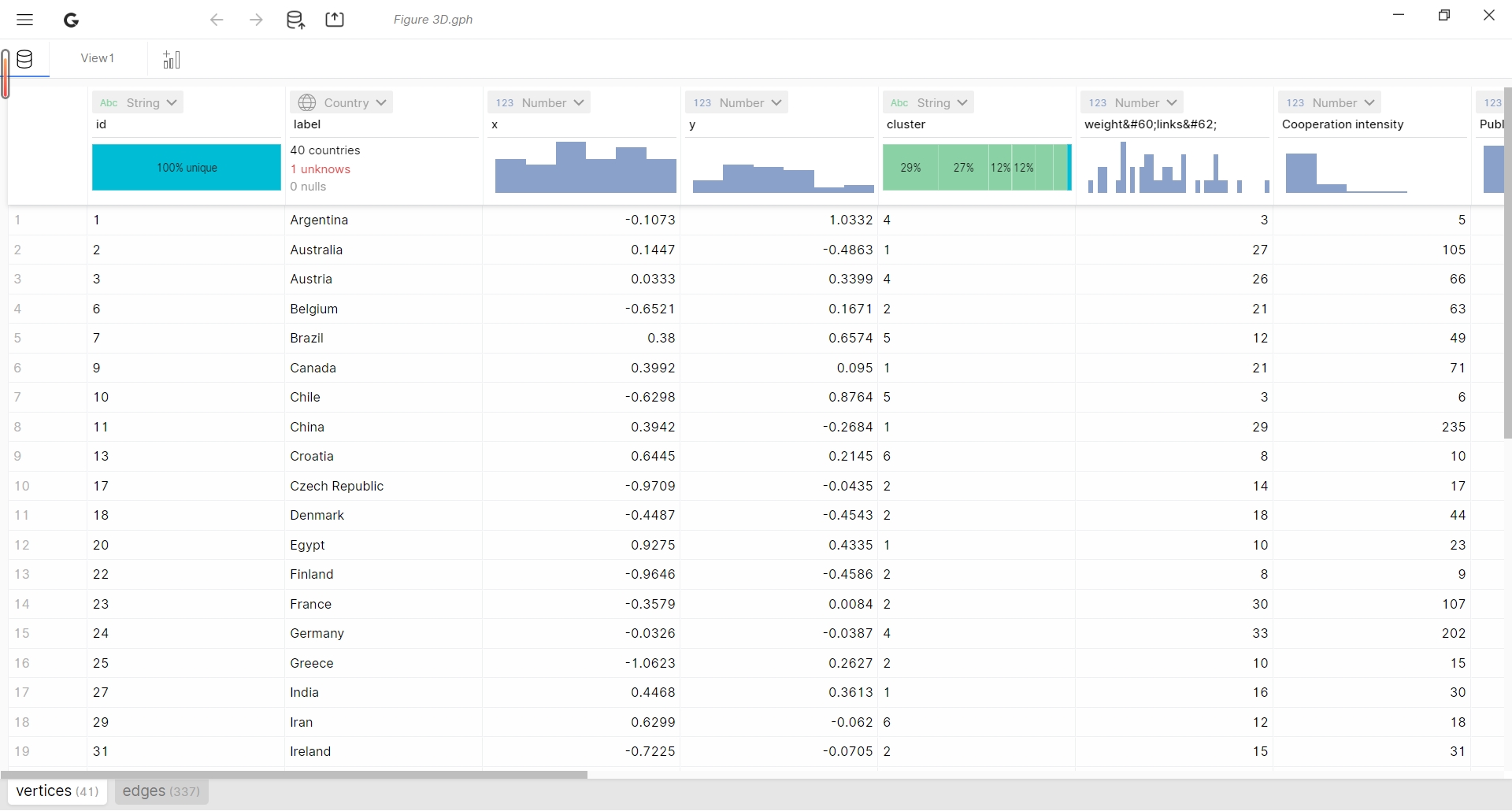
Supplementary Figure 1H：Parameters 1 for Top 30 countries visualization


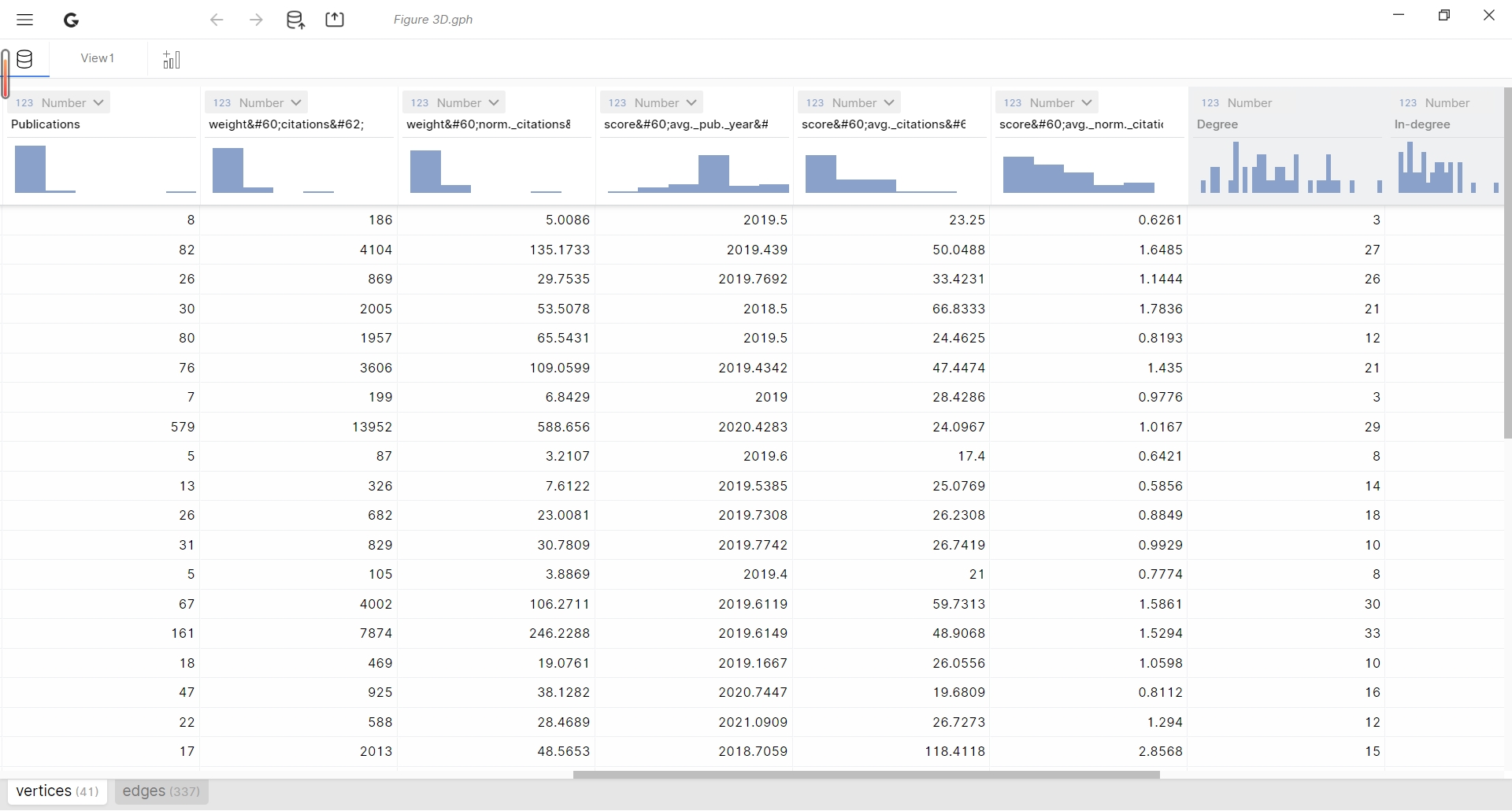
Supplementary Figure 1I：Parameters 2 for Top 30 countries visualization


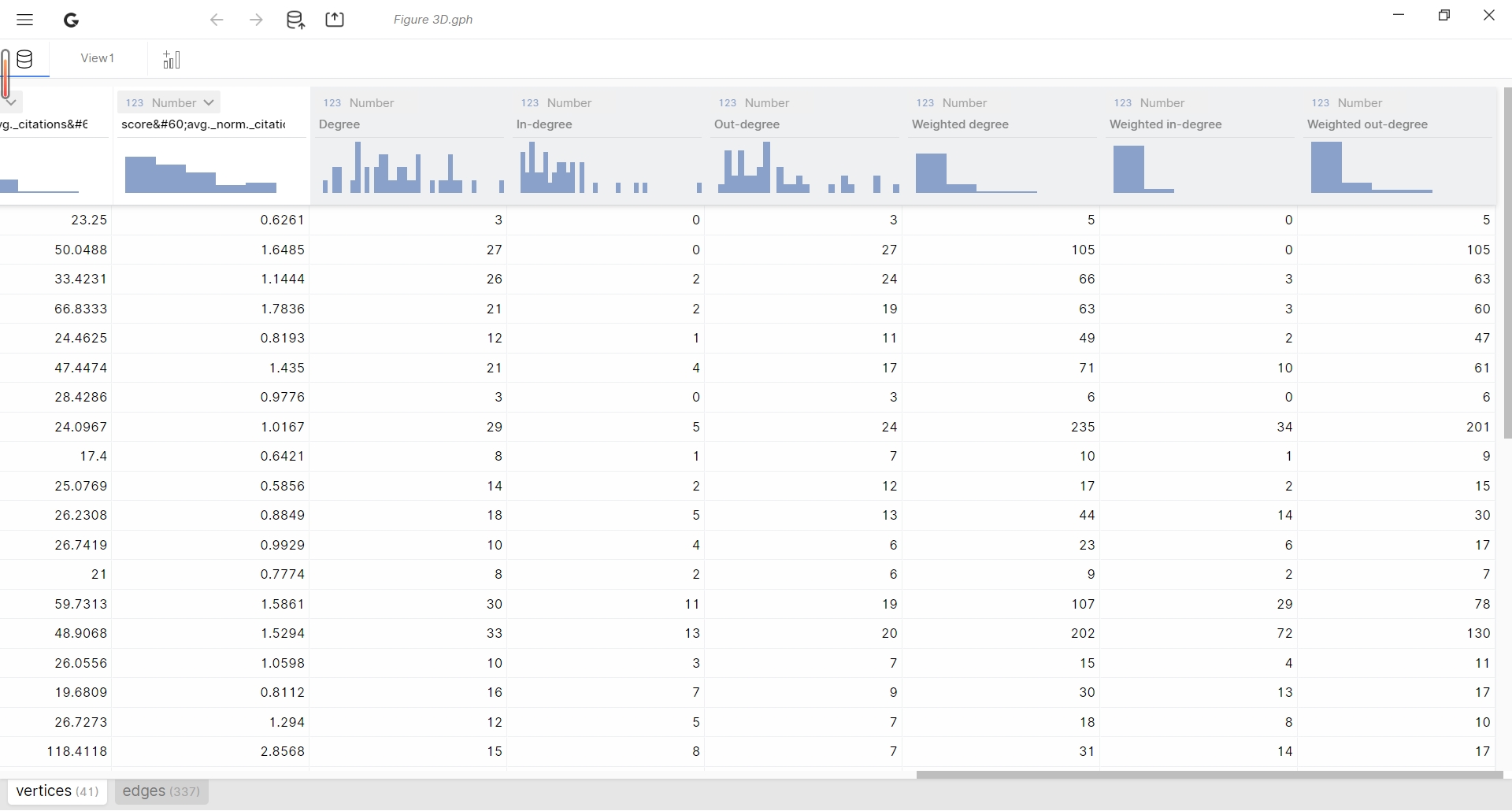


Supplementary Figure 1J：Parameters 3 for Top 30 countries visualization

Figure 4A and Figure 4C were created using CiteSpace (6.3.R1), with specific parameters provided in Supplementary Figure 2A and 2B. Figure 4B and Figure 4D were obtained respectively from publications and frequency run by CiteSpace.


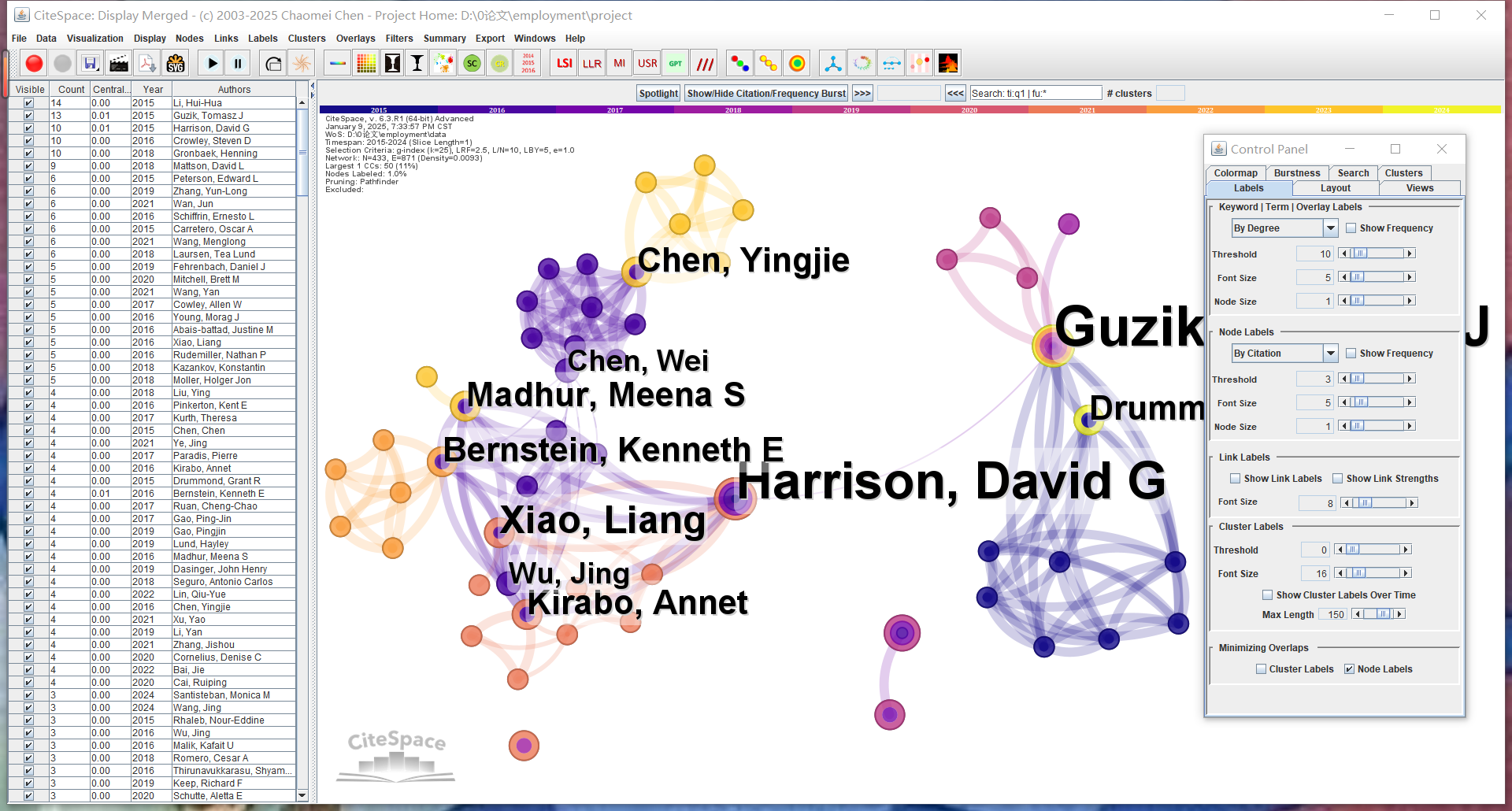


Supplementary Figure 2A：All parameters for visualization of co-occurrence of authors.


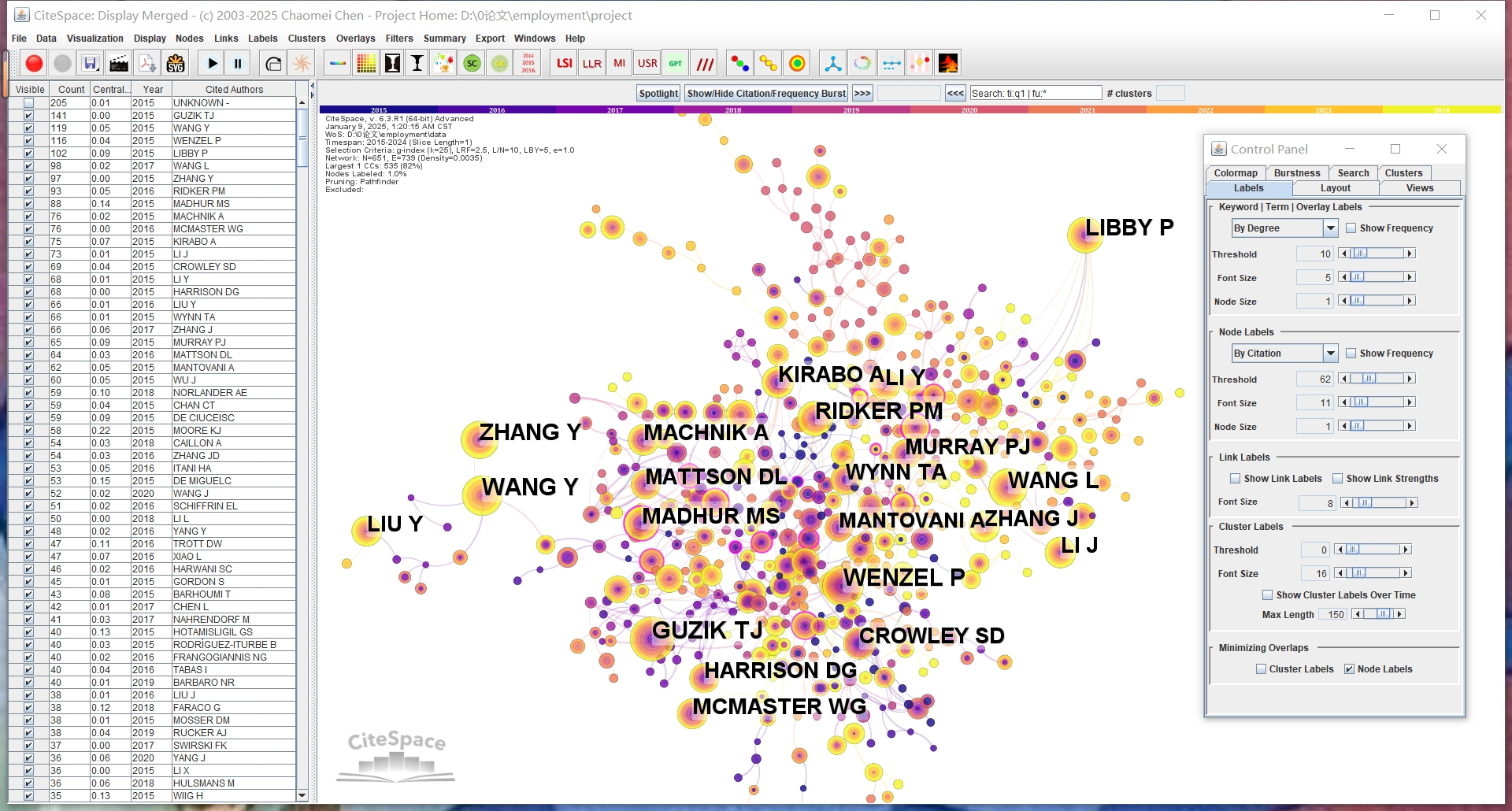


Supplementary Figure 2B：All parameters for co-citation visualization map of authors.

Figure 5 was created using CiteSpace (6.3.R1), with specific parameters provided in Supplementary Figure 3.


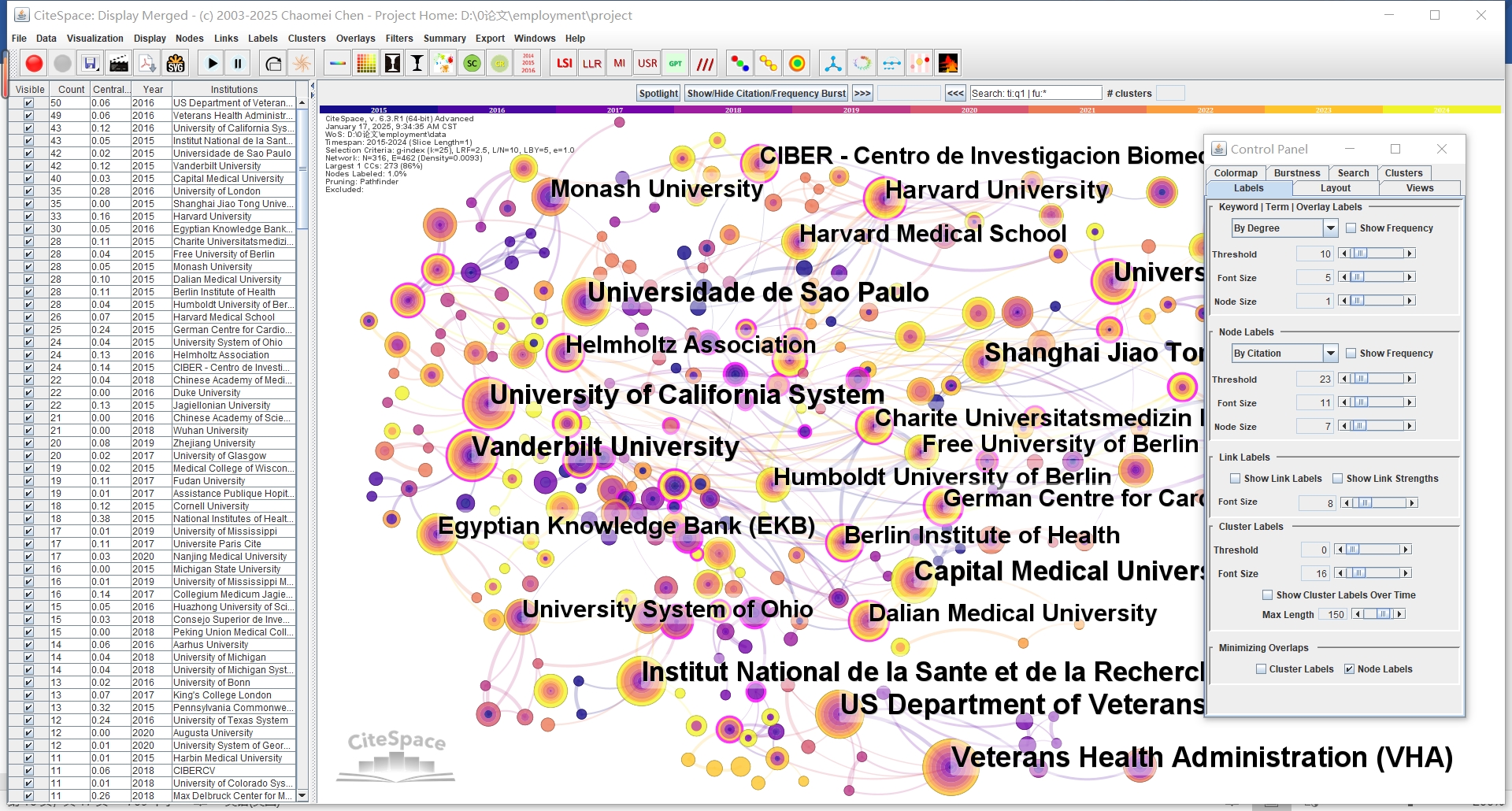


Supplementary Figure 3：All parameters for visualization of cooperation networks among institutions

Figure 6A was created in VOSviewer 1.6.20, while Figure 6B and Figure 6C were created using CiteSpace (6.3.R1), with specific parameters provided in Supplementary Figure 4.


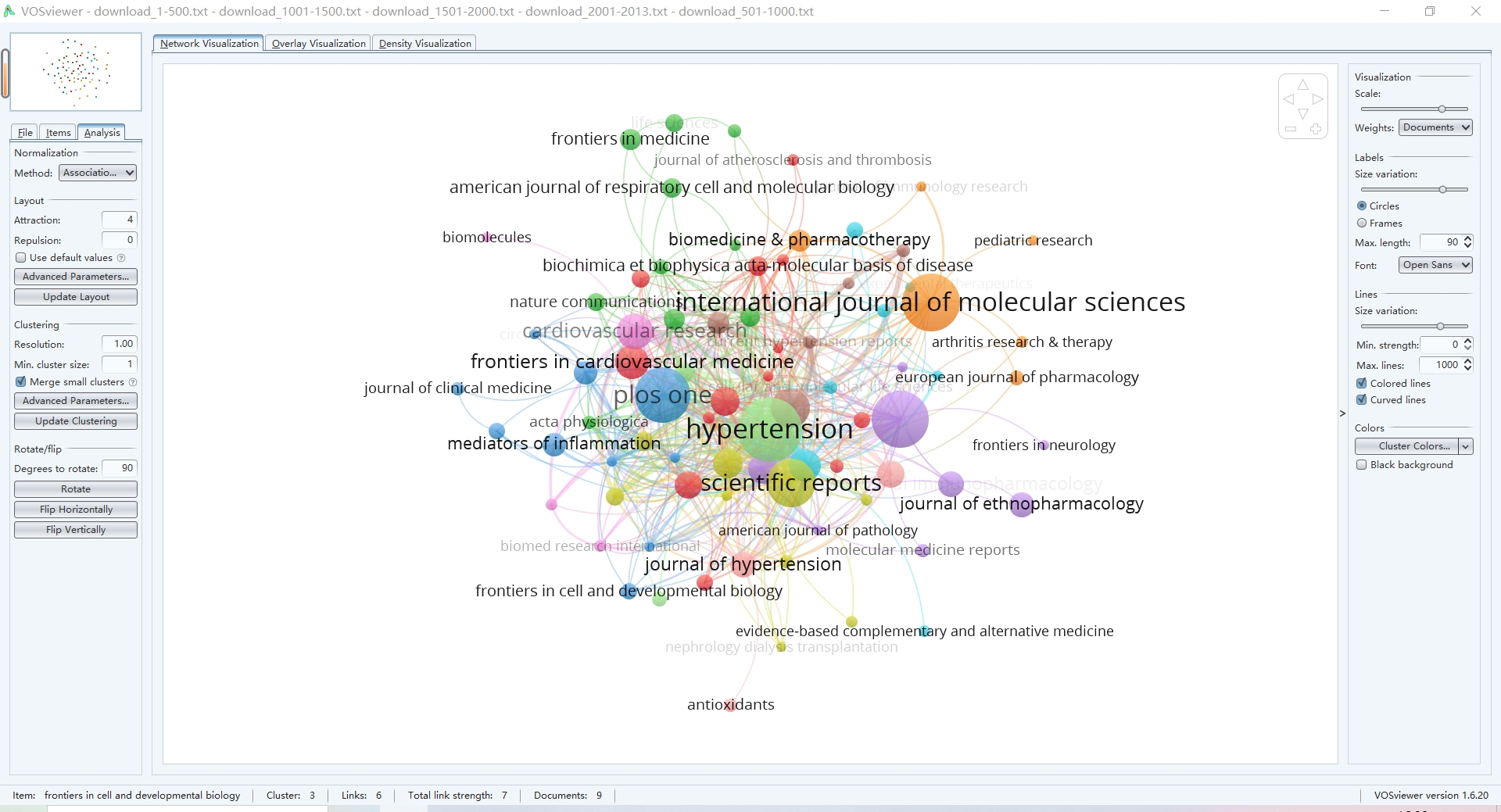


Supplementary Figure 4A：All parameters for network visualization of source journals.


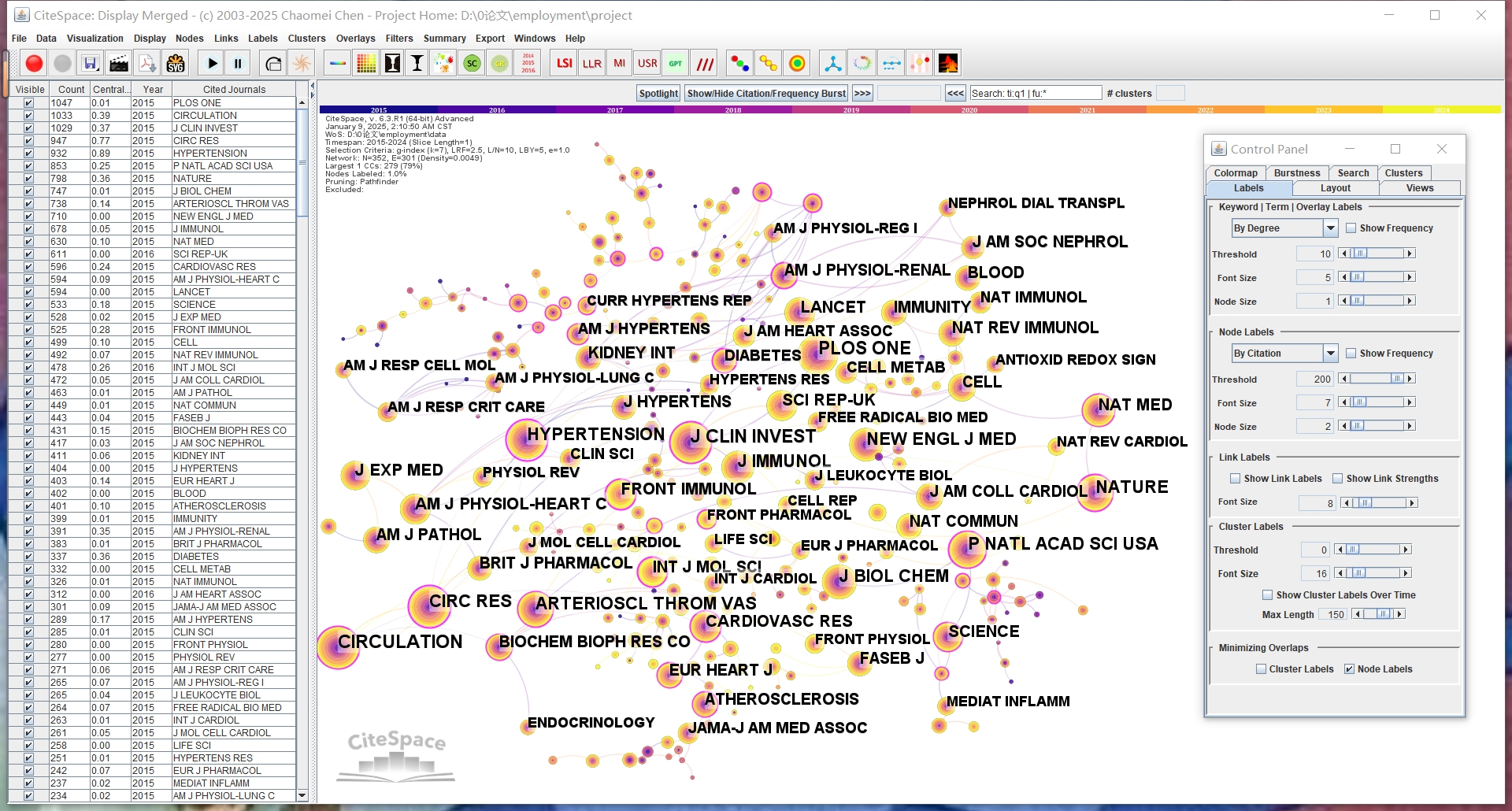


Supplementary Figure 4B：All parameters for Visualization of cited journals.


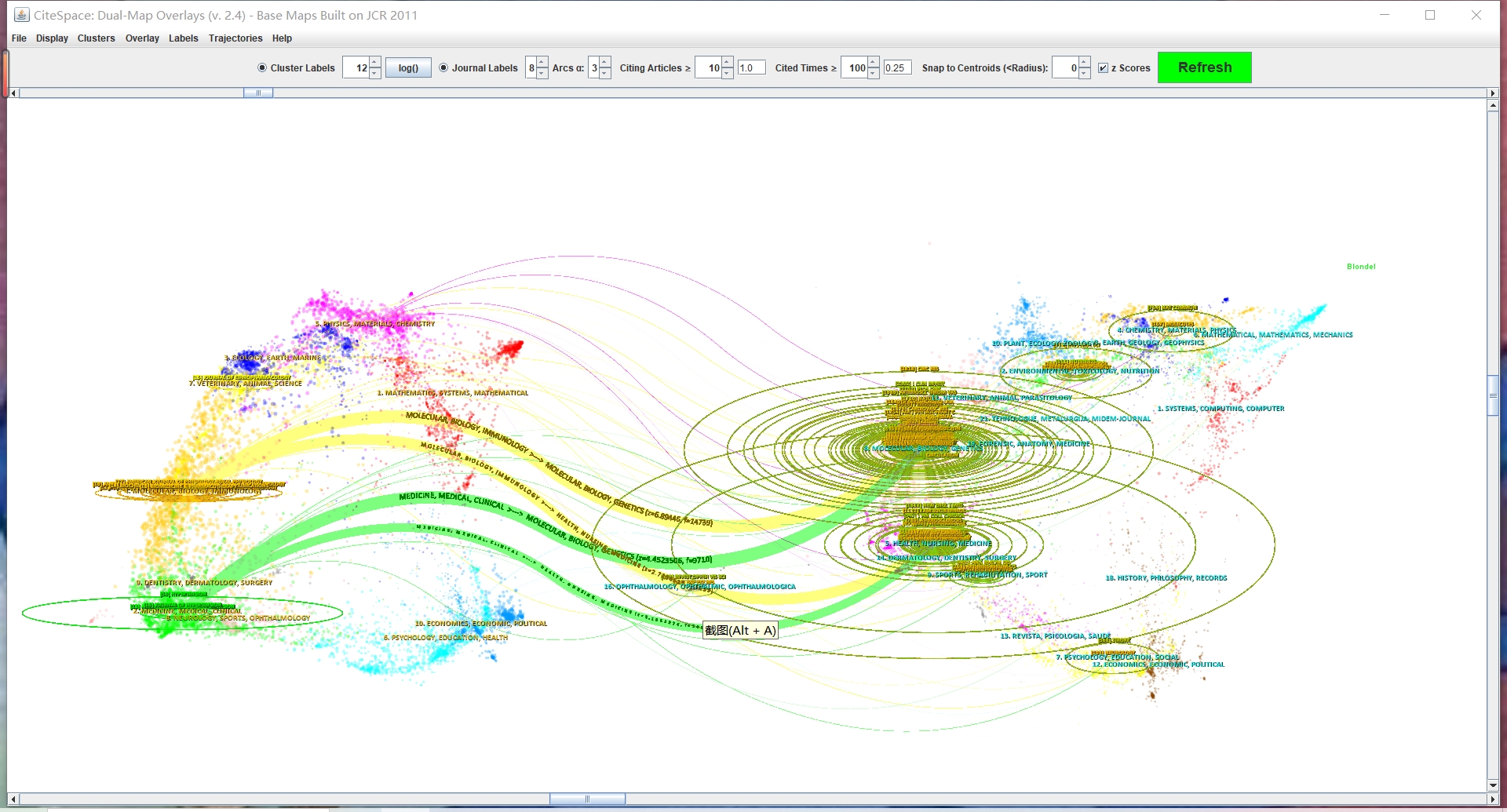


Supplementary Figure 4C：All parameters for the dual-map overlap of journals.

Figure 7A and Figure 7B were created using VOSviewer 1.6.20, with specific parameters provided in Supplementary Figure 5A and Figure 5B. Figure 7C, Figure 7D, Figure 7E and Figure 7F were created using CiteSpace 6.3.R1, with specific parameters provided in Supplementary Figure 5C-5F.


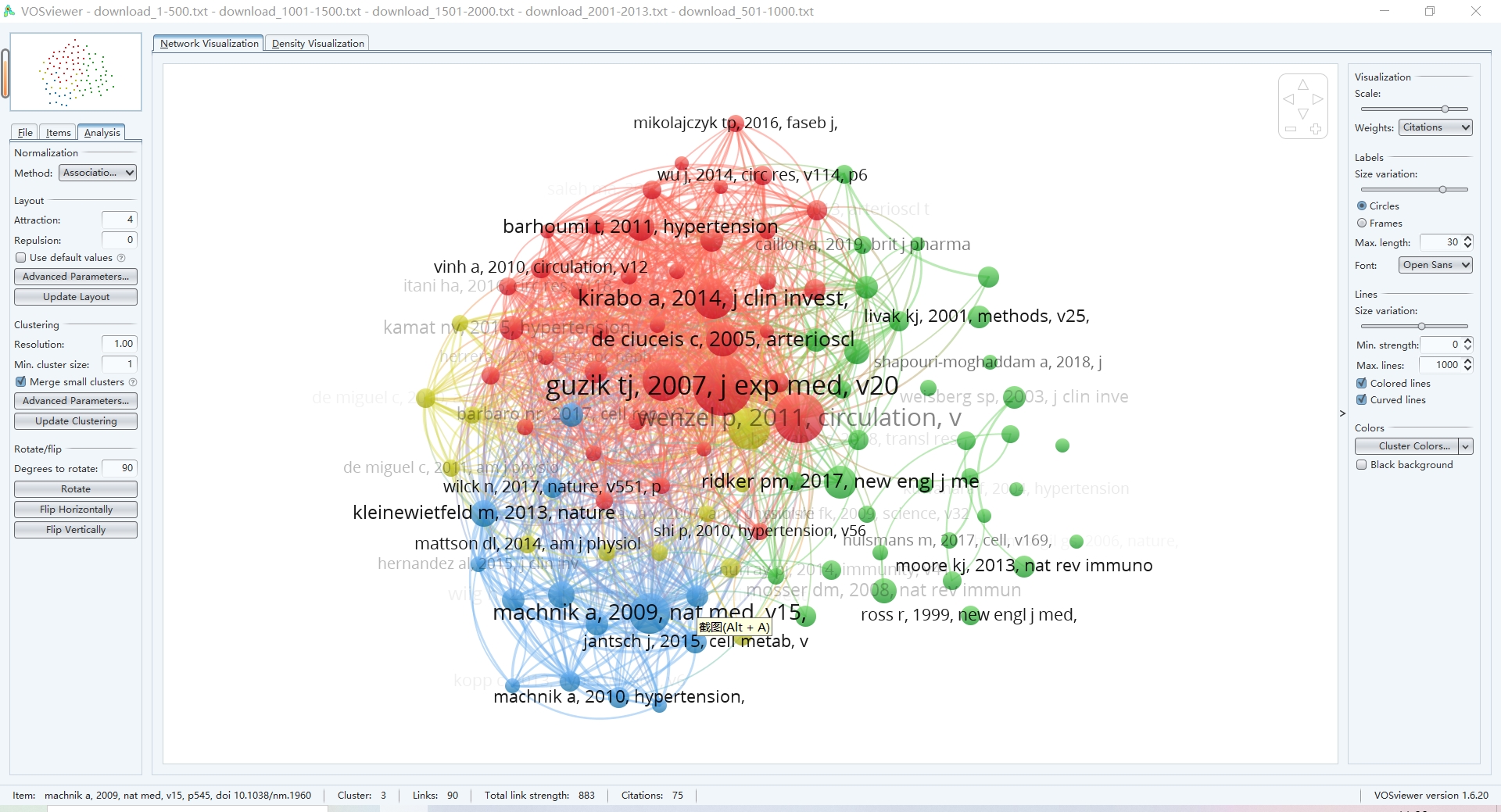


Supplementary Figure 5A：All parameters for references co-citation network.


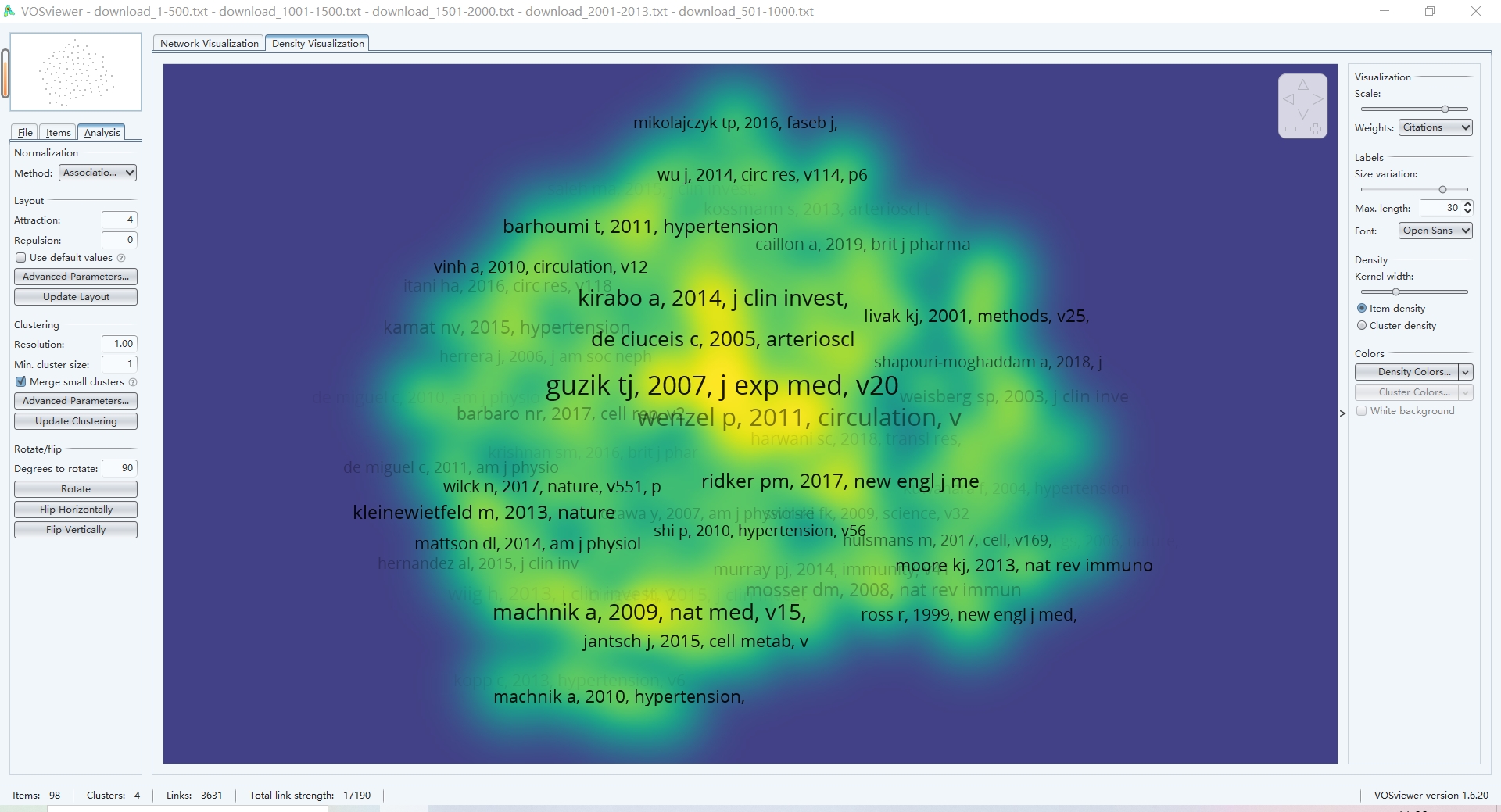


Supplementary Figure 5B：All parameters for density visualization of co-cited references.


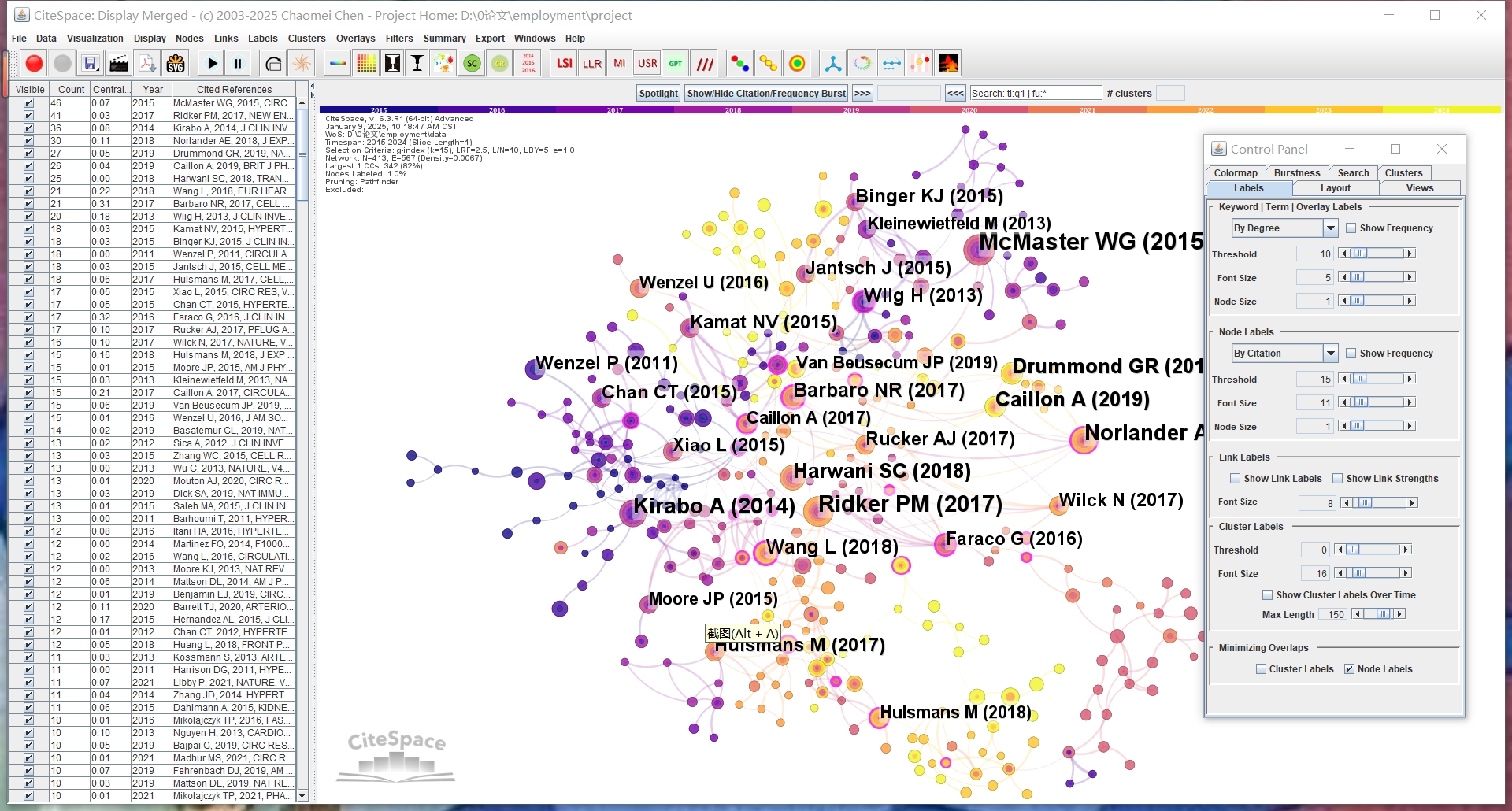


Supplementary Figure 5C：All parameters for references co-citation network.


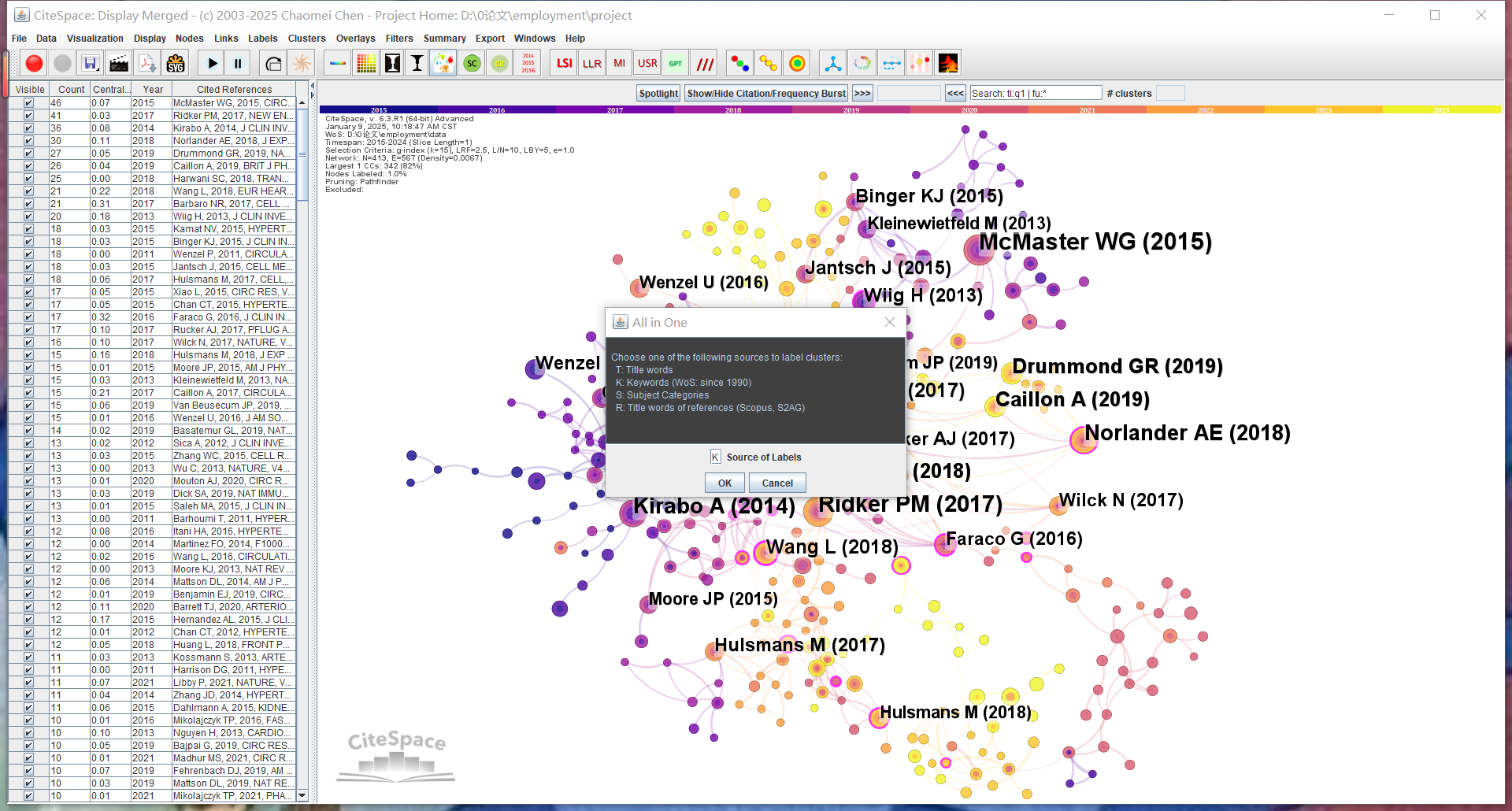


Supplementary Figure 5D：All parameters for references co-citation network.


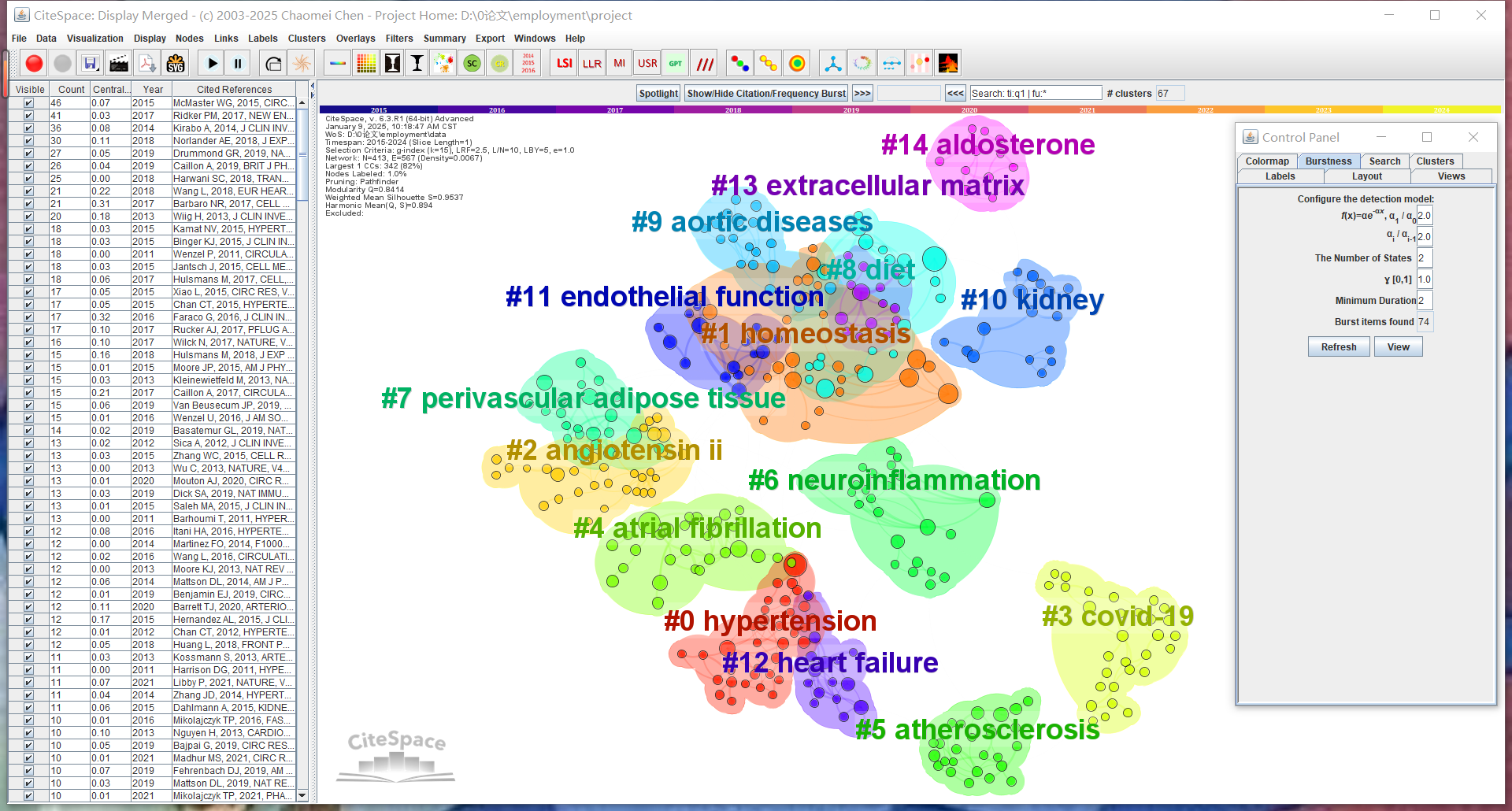


Supplementary Figure 5E：All parameters for references co-citation network.


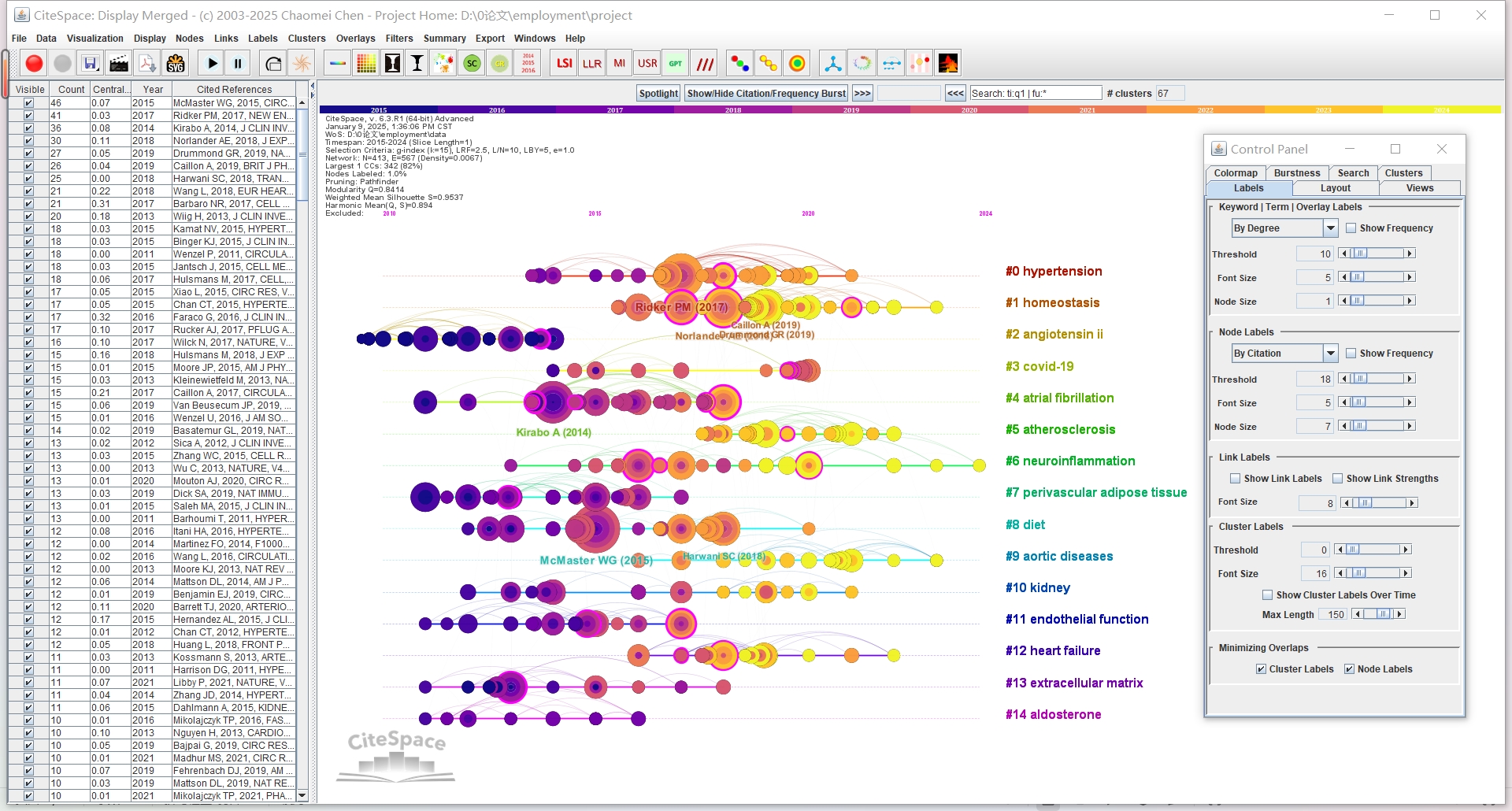


Supplementary Figure 5F：All parameters for references co-citation network.

Figure 8A and Figure 8B were created using VOSviewer 1.6.20, with specific parameters provided in Supplementary Figure 6A and Figure 6B. Figure 8C and Figure 8D were created using CiteSpace 6.3.R1, with specific parameters provided in Supplementary Figure 6C and Figure 6D.


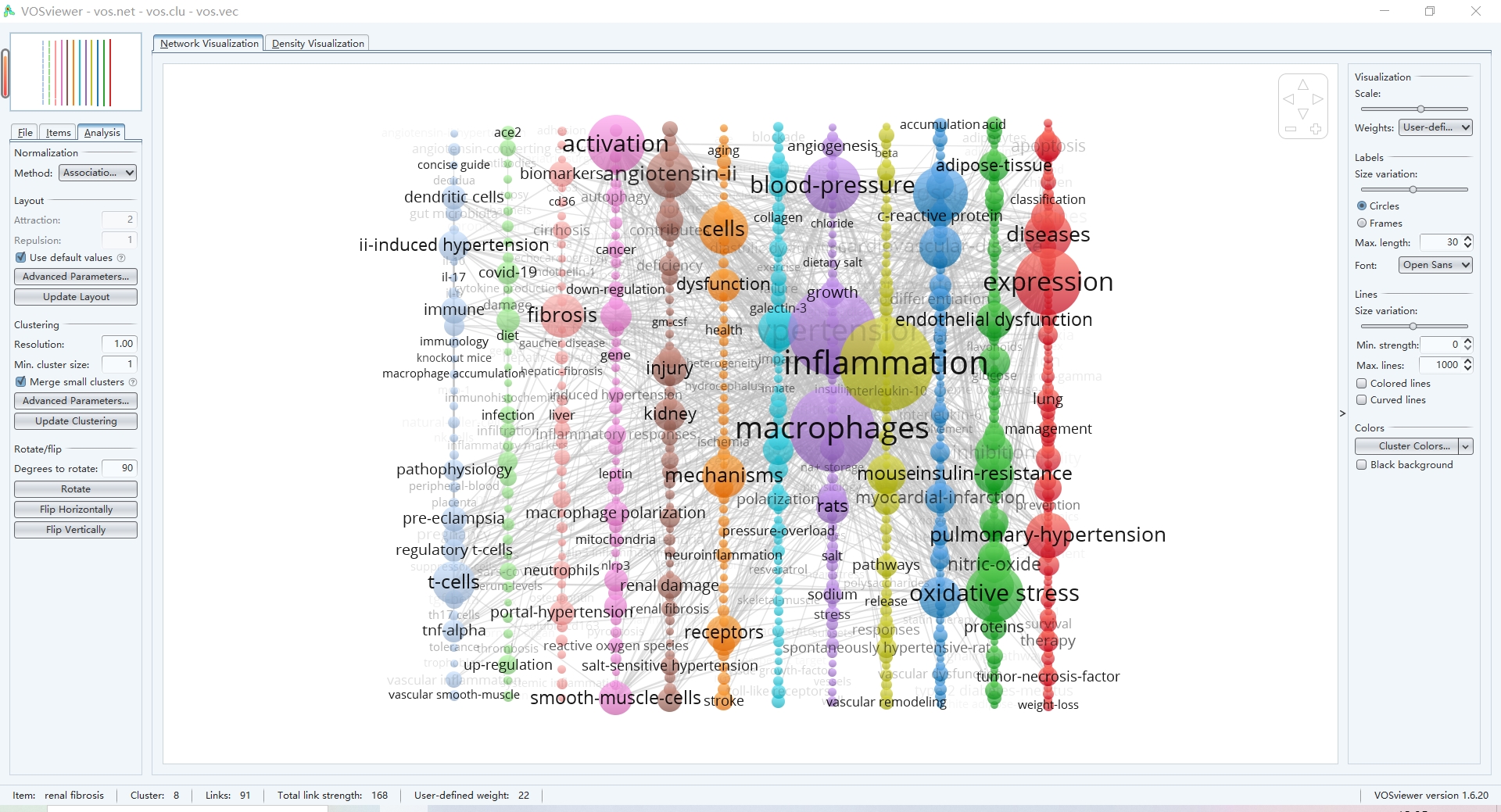


Supplementary Figure 6A：All parameters for keywords clusters.


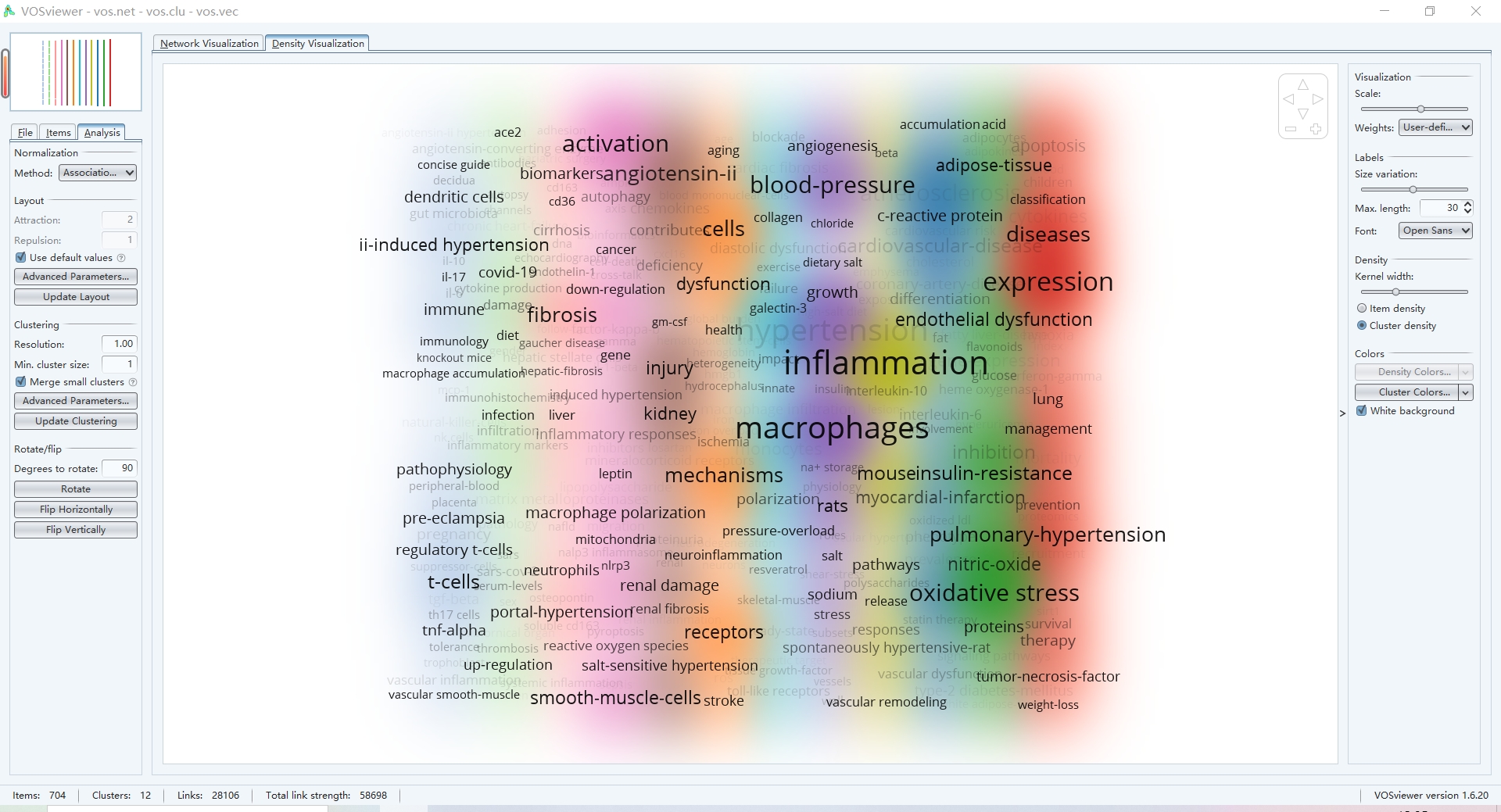


Supplementary Figure 6B：All parameters for density visualization of keywords clusters.


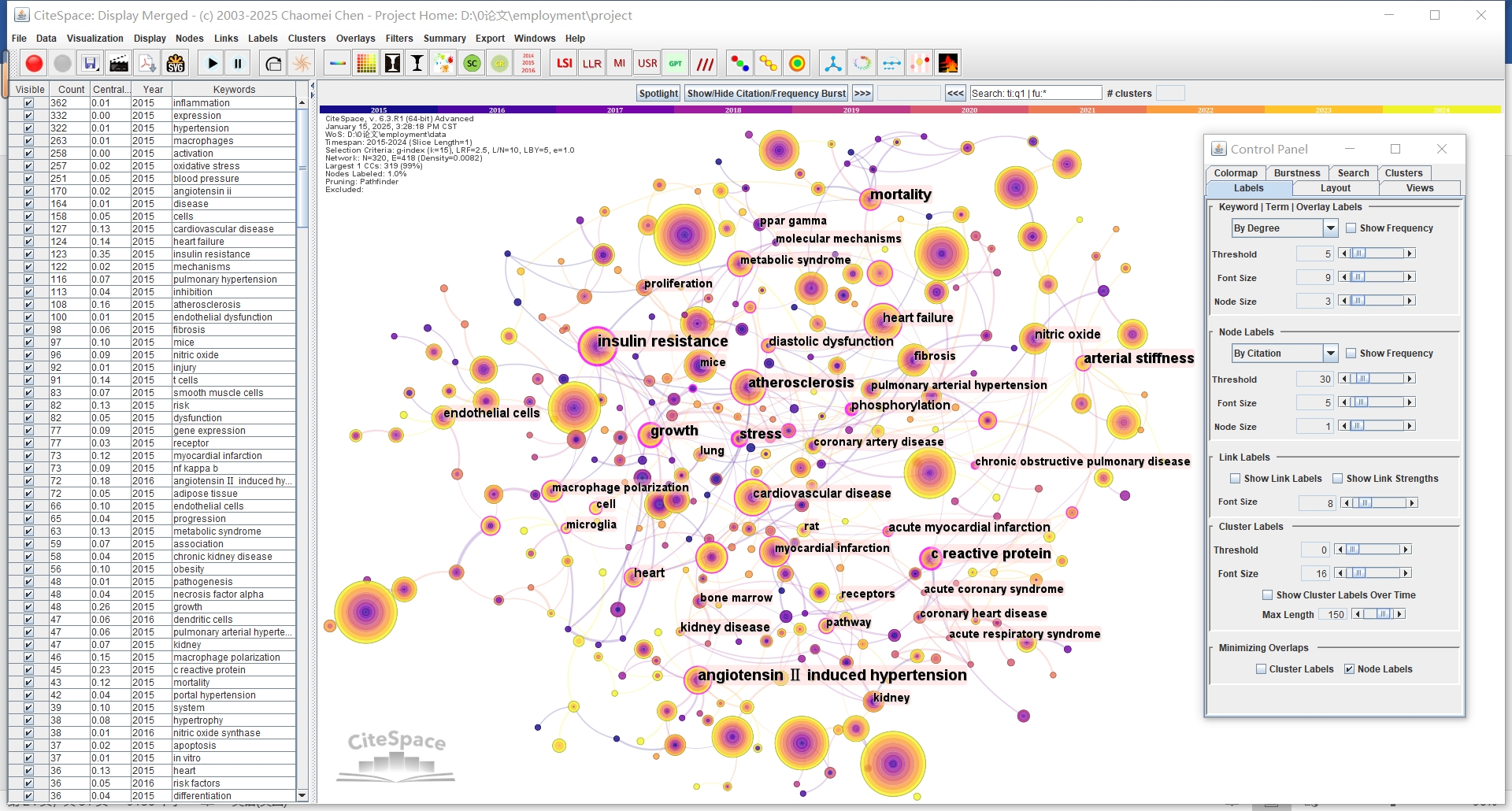


Supplementary Figure 6C：All parameters for keyword visualization.


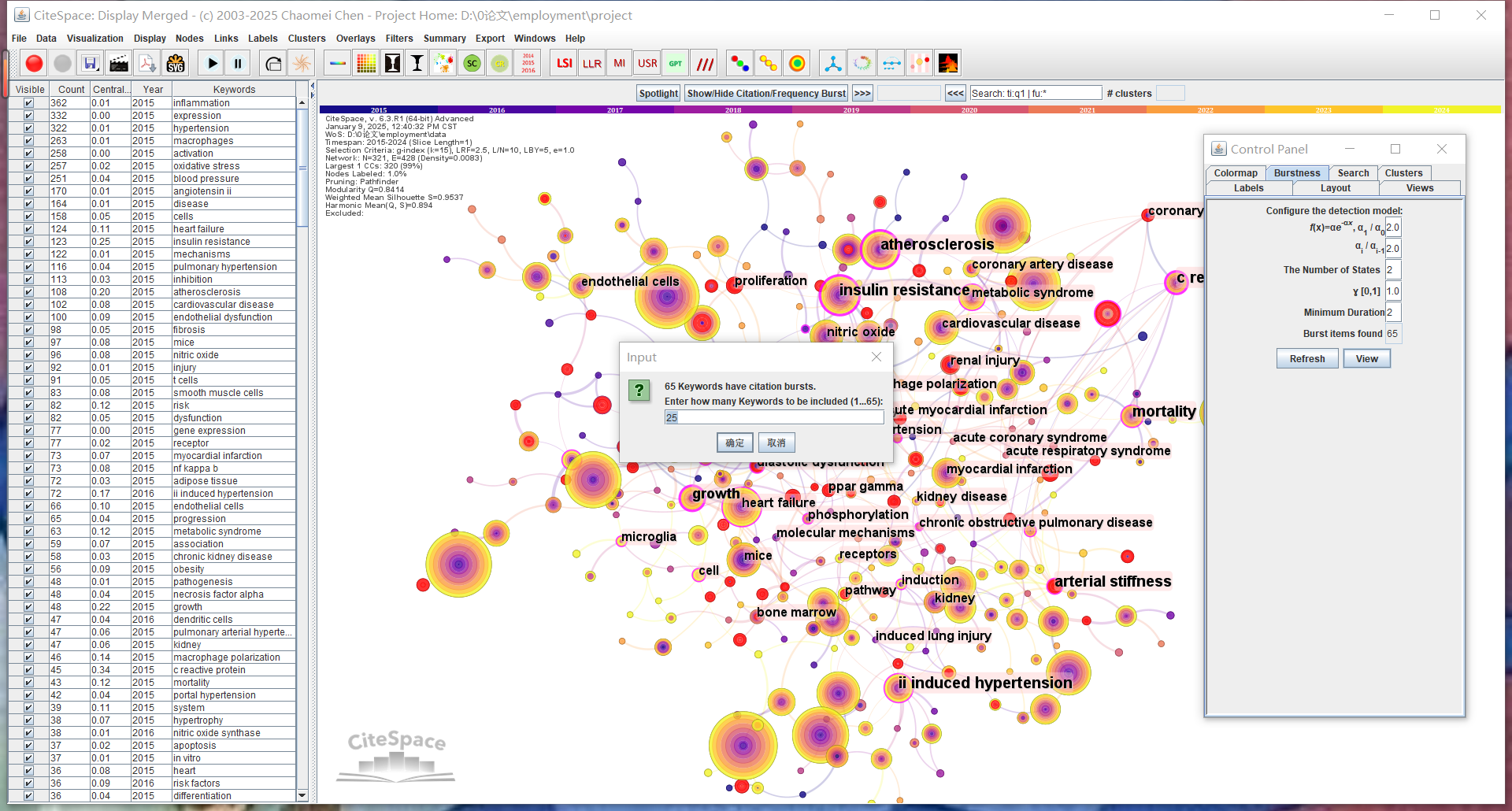


Supplementary Figure 6D：All parameters for top 25 keywords with the strongest citation bursts.
